# Change in risk of breast cancer after receiving hormone replacement therapy by considering effect-modifiers: a systematic review and dose-response meta-analysis of prospective studies

**DOI:** 10.18632/oncotarget.20154

**Published:** 2017-08-11

**Authors:** Kang Wang, Feng Li, Li Chen, Yan-Mei Lai, Xiang Zhang, Hong-Yuan Li

**Affiliations:** ^1^ Department of the Endocrine and Breast Surgery, The First Affiliated Hospital of Chongqing Medical University, Chongqing Medical University, Chongqing, 400016, China; ^2^ Department of Neurosurgery, The Second Affiliated Hospital of Chongqing Medical University, Chongqing Medical University, Chongqing, 400016, China

**Keywords:** breast cancer, hormone replacement therapy, estrogen-alone therapy, estrogen plus progestin therapy, body mass index

## Abstract

We synthesize the current literatures and use the power of meta-analysis to examine trends on association between hormone replacement therapy (HRT) and the risk of breast cancer (BC). We performed a comprehensive literature search using PubMed, EMBASE, and Web of Science from their inception until Jan 2017. Prospective studies that provided adjusted risk estimates of HRT and BC risk were eligible. Categorical and dose-response meta-analyses followed the PRISMA were conducted using random effects model and restricted cubic spline model, respectively. Forty-seven publications from thirty-five unique studies were included, involving 3,898,376 of participants and 87,845 of BC cases. Compared with non-users, RR for current estrogen-only therapy (ET) users was 1.14 (95% confidence interval (CI) = 1.05–1.22), and for per year increases was 1.02 (95% CI = 1.02–1.02). Moreover, RR for current estrogen plus progestin therapy (EPT) users was 1.76, (95% CI = 1.56–1.96), and for per year increases was 1.08 (95% CI = 1.08–1.08). Dose-response analyses revealed 8–10 years’ onset peaks, and indicated residual increased BC risk remained after stopping use of ET regimen rather than for EPT. Effect-modifiers like BMI, duration of use, race/ethnicity, routes of administration were recognized. In Conclusions, current use of EP or EPT and ever use of tibolone are associated with an elevated risk of BC. Compared with slim HRT users and non-users, lower BC risks were found among overweight/obese HRT users and former EPT users, respectively. Both ET and EPT users are associated with higher risk of lobular BC than ductal BC, and more ER-positive than negative BC cases were detected among EPT users.

## INTRODUCTION

After increasing breast cancer (BC) risk among hormone replacement therapy (HRT) users was detected by Women's Health Initiative (WHI) Randomized Clinical Trial (RCT) in 2002 [1], HRT were challenged by International Agency for Research on Cancer (IARC) [2, 3]. This cancer accounts for leading incidence as well as second cause of death in women cancers [4]. Overwhelming evidence from current decade showed a striking disparity between estrogen-alone therapy (ET) and estrogen plus progestin therapy (EPT) regimens in incidence of BC [5–12], recommending that scholars should not combine any HRT regimens when examining BC risk [13].

Although the relationship between EPT use and elevated BC risk has been well established, newly developing perspectives and issues were difficult to interpret according to prior evidences [12, 14, 15]. For instance, the tendency of BC risk changes over time when extending to a long-term use (> 10years) of EPT, and whether increased EPT-associated BC risk drops after stopping it [7, 9]. Additionally, some effect-modifiers and interactions were mentioned for precise risk assessment, such as age [16], race/ethnicity [13], body mass index (BMI) [5, 7, 9, 17–21], progestin type [5, 22–24], route of administration [5, 22, 25], mode of EPT combination [5, 18, 21–24, 26], characteristics of BC [11, 18, 19], and gap time between menopause and starting use EPT [19, 27]. Similarly, the discrepancy in BC risk was observed among ET users according to these inconsistent factors. Most observational studies [5, 8, 9, 20, 26, 27] are characterized as inadequate for weighing this risk because of these potential effect-modifiers.

Given the comparable elevated risk with unopposed estrogen use in development endometrial cancer [28], ET is usually applied to subjects undergone hysterectomy. Interestingly, low-level endogenous estradiol [29] or estrogen antagonist (e.g. tamoxifen [30], oophorectomy [31] and menopause [15].) had been certified as BC risk reduction factors, indicating that estrogen exerts a promotional effect on developing BC, while there has been no verdict regarding inconsistent results shown by epidemiological studies. The WHI RCT [32] showed no additional BC risk among 5,310 women receiving 0.625mg/day conjugated equine estrogens(CEE) compared with placebo group, instead, BC incidence is lower but without significant difference detected. Nevertheless, considering increased stroke risk and no overall favorable risk to benefit ratio, the trial had to be ended after 7.2 years’ intervention [32]. When followed-up duration extended to more than 10 years after initial intervention, this potential benefit not only sustained but also became notable throughout the early post-intervention phase [7]. In contrast, some large, prospective, population-based observational studies consistently indicated an association between ET use and increased risk of BC, such as Million Women Study [27], Nurses’ Health Study [33], California Teachers Study [18] and a Danish cohort [23], raising uncertainty on the magnitude of ET-related BC risk. There were inevitable potential biases as misclassification of actual use [34], menopausal age [6] and unsatisfied adherence to treatment may apparently lead to inconsistent results. Meanwhile, BMI profile should be comparable when discussing this ambiguous correlation, which is a critical factor for ‘estrogen paradox’ [35, 36].

We carried out a systematic review with dose-response meta-analyses of different HRT regimens and subsequent BC in order to identify the shape of utility and withdrawal associated time-response and quantify the precise outcomes and the effects of potential interactions on HRT-related BC.

## MATERIALS AND METHODS

### Literature search

This systematic review with meta-analysis was conducted and reported in accordance with PRISMA guideline [37]. A comprehensive literature search was performed using PubMed, EMBASE, and Web of Science from their inception until Jan 2017 (date last searched), which included truncated free text and explored mesh terms. The detailed search strategies were showed in Supplementary Table 1. To avoid missing studies, we manually checked the reference lists of previous reviews. No attempt was made to identify unpublished reports. If necessary, the original authors were contacted to obtain extra information via e-mails.

### Study selection

RCTs, prospective cohort studies and nested case-control studies that assessed HRT (i.e. EPT, ET, PT) as exposure variables and BC as an outcome and supplied risk estimates with 95% confidence interval (CI) were eligible for categorical analyses. To conduct dose-response analyses, quantitative exposures (duration or time from quitting of HRT use and BMI) had to be available additionally, and BC cases and person-years in each quantitative category should be reported. We employed the studies with longer followed-up and more detailed data under the condition that multiple publications reported the same database. Nevertheless, some overlapping publications with detailed information, not for main analysis, were exploited for subgroup analyses. Two investigators (L.C. and F.L.) independently screened titles and abstracts to identify the potentially suitable publications, then they evaluated these relevant articles based on full-texts reviewing. Any discrepancies were solved through consensus.

### Data extraction and quality assessment

Data extraction was performed by one investigator (F.L.), and was then checked independently for the accuracy by another investigator (L.C.). The following information was extracted: first author, year of publication, study location, sample size, BC cases, mean age, exposure or interventional variables, mean follow-up duration, BC assessment and maximally adjusted risk estimate with corresponding 95% confidence interval (CI) and adjustment factors.

We assessed the quality of identified studies using the Newcastle-Ottawa quality assessment scale (NOS) [38]. To evaluate its 3 aspects (selection, comparability, and outcome), nine stars could be awarded to each study at most (4 stars for selection; 3 stars for comparability; 2 stars for outcome). The quality of studies ranks as low quality (below 3 stars), moderate quality (4–6 stars), high quality (7–9 stars). Any disagreements on the results of data extraction and quality assessment were resolved by further discussion.

### Statistical methods

We performed categorical and dose-response meta-analysis, and random effects model was used to pool risk estimates [39]. Relative risk (RR) was adopted to evaluate the association between HRT use and the risk of BC. Hazard ratio (HR) [6–9, 40–42], odds ratio (OR) [43], incidence rate ratio (IRR) [21, 44] and standardized incidence ratio (SIR) [22] were considered as equivalent to RR simultaneously. Maximally adjusted RRs were employed to yield summary results, which were calculated using the average of natural logarithm RRs of eligible studies, and weighted by the inverse of the variance, whereas unadjusted RRs from the RCT [7] was also included.

Categorical meta-analysis was conducted by pooling basic classification results involving HRT regimens (i.e. ET, EPT and other HRT regimens) at different use statuses (i.e. current, former). The reference category was individuals never using HRT, and exposure group was current, former and ever HRT users responsible for above classifications, depending on studies. When categorical results were stratified by age [40], pathology [20, 45, 46], subjects undergo operations [33] and duration of use [9], we combined subgroup specific outcomes using fixed effects model to generate an uniquely categorical effect.

The random-effect dose-response on account of generalized least squares trend estimation proposed and developed by Orsini and Greenland [47, 48] was used to explore RR with 95% CI for per 1 unit increase from linear trend on association between duration of HRT use or quitting (year) or BMI (kg/m^2^) among HRT users and the risk of BC, respectively, and a goodness-of-fit *chi^2^* with *P_goodness-of-fit_* was calculated to test the suitability of the Restricted cubic spline models were applied to non-linear dose-response analysis using spline transformations with three knots at the 10th, 50th and 90th percentile [49, 50], and a *P_non-linearity_* value for curve linearity or non-linearity was calculated by testing the null hypothesis that the estimated value of the second spline is equal to zero [49]. The models were based on specific exposure level, distribution of cases, person-years and the RRs with 95% CIs for at least three quantitative categories. We formulated means or medians of the quantitative categories as each exposure level, if not reported in studies, the estimated midpoint must be available. Furthermore, if the highest category was open ended, midpoint of whose category was assigned at same adjacent with the lower boundary. When the lowest category was open-ended, we set the lower boundary to zero. In particular, 15 kg/m^2^, 18.5 kg/m^2^ and 40 kg/m^2^ were employed as lowest bound and highest bound supposing that studies reported results by WHO categories of underweight (< 18.5 kg/m^2^), normal (18.5–25 kg/m^2^) and obesity (> 30 kg/m^2^), respectively. Of BMI categories with non-zero level as reference [6, 7, 18, 20, 21, 45], we adopted method of Hamling and colleagues [51] to convert risk estimates when reference group was not the lowest category. Meanwhile, when we explored dose-response associations of time since last HRT use and BC risk, current HRT use was considered as reference group after similar transformations. For some studies, whose original researchers did not report person years by exposure level [8, 41, 52], we approximately derived such data from follow-up duration and the number of participants at each quantitative category.

Subgroup analysis and meta-regression [53] with a *P_interaction_* were performed stratified by duration of current use, time since last HRT use, baseline characteristics of participants (i.e. age at study entry, race/ethnicity, BMI), types of hormone component, route of administration (i.e. oral, virginal, transdermal), mode of EPT combination (i.e. sequential, continuous), characteristics of BC (i.e. stage, histology, ER/PR status). Particularly, age and race/ethnicity were study-level factors which were identified by population characteristics, instead the rest of variables deriving from categorical outcomes in primary studies were employed to conduct precise subgroup analyses. In addition, we respectively presented dose-response analyses results on duration of HRT use and BC risk with 25 kg/m^2^ and 5years as the boundary values adopt by most studies to assess potential interaction effects from BMI and gap time.

To identify potential heterogeneity, we calculated Q statistic (*P_Heterogeneity_* < 0.10 suggesting statistically significance) and the I^2^ statistic [54], whose values of 0, 25, 50, and 75% are regarded as no, low, moderate and high heterogeneity. Sensitivity analyses were conducted by ignoring a single study in turn. Small study or publication bias was assessed by Begg rank correlation test [55] and Egger linear regression test [56]. All analyses were conducted using STATA version 13.0 (Stata Corp, College Station, TX). *P* < 0.05 was considered statistically significant. All statistical tests were two-sided.

## RESULTS

### Literature search and study characteristics

**Figure 1:**
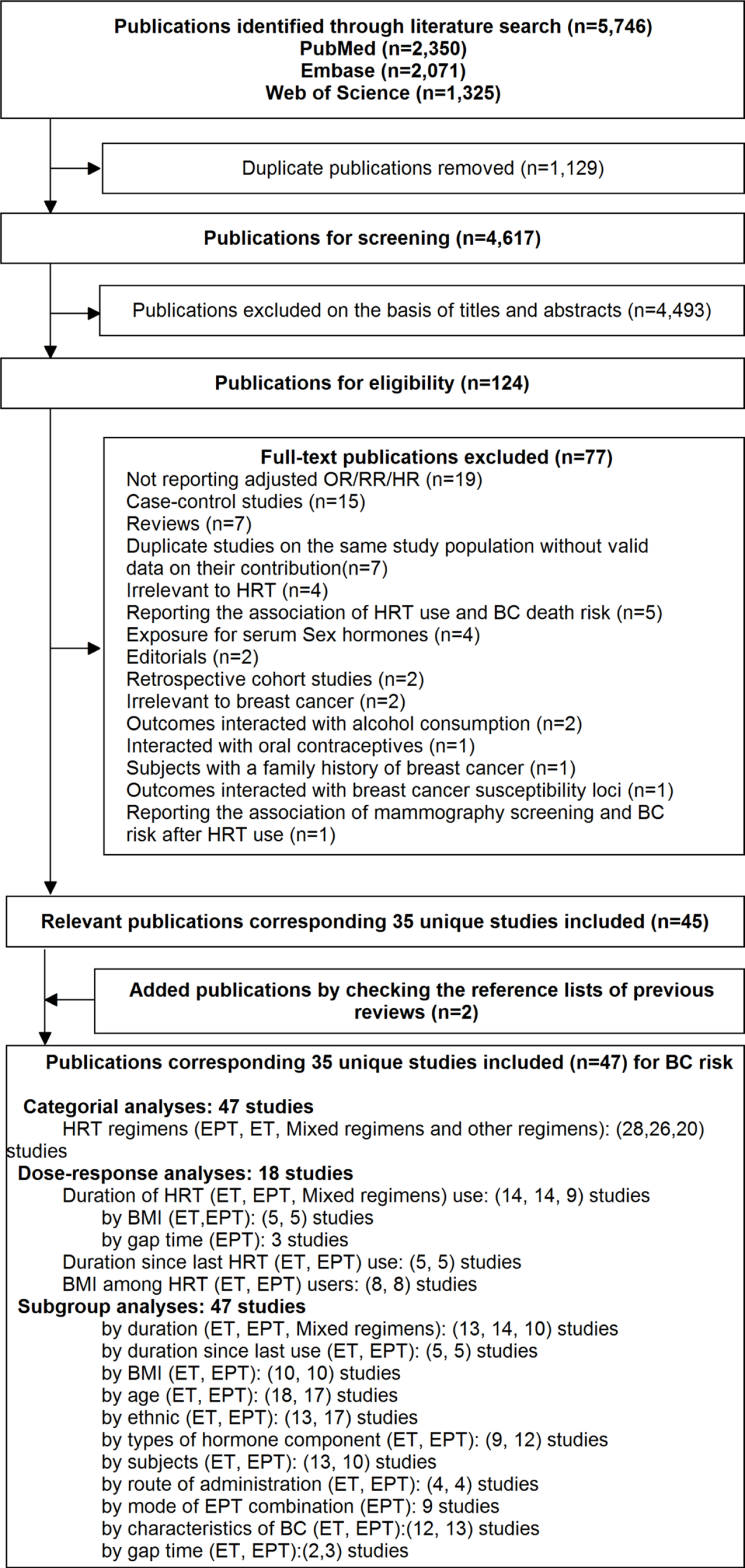
The flowchart of selecting eligible studies

Figure 1 shows process and results from the literature search. 2350 articles were identified from the PubMed database, 2071 articles from the EMBASE database, and 1325 articles from the Web of Science database. A total of 4,617 studies remained after exclusion of duplicates, and we obtained 124 potential relevant studies by screening titles and abstracts. After full text reviewing, we excluded 79 studies for detailed reasons showing in Supplementary Table 2. Additional 2 [16, 17] studies were eligible for inclusion in process of reviewing reference lists of prior reviews. At last, forty-seven publications from thirty-five unique studies were included, of which the characteristics shows in Supplementary Table 3. Some studies were reported using multiple publications, while we selectively included them for more comprehensive outcomes. Seven eligible publications [1, 7, 13, 32, 57–59] reported results from WHI RCT and its subsequent observational study. Four eligible publications [9, 60–62] reported results from E3N cohort, and three [11, 27, 34] reported on Million Women Study. Nurses’ Health Study cohort was effectively reported by two publications [19, 33].

Thirteen studies were from Northern Europe [5, 22–24, 41, 42, 44, 46, 52, 63–66], 12 studies from USA [8, 17–21, 26, 43, 67–70], 3 studies from UK [6, 8, 27], 3 studies from France [9, 25, 71], 1study from Japan [40], 1study from Canada [72], 1study from Netherlands [73] and a [16] international study respectively. The average follow-up among prospective studies changed from 2.6 years [34] to 16.5 [19]. The sample size including the number of exposure and non-exposure (or placebo and intervention) population ranged from 1,334 [43] to 1,129,025 [27], including 3,898,376 of participants and 87,845 BC cases in total. Ascertainment methods of HRT exposure varied from studies, and most studies employed questionnaires to interview or mail participants [6, 8, 9, 16, 17, 20, 21, 23–26, 41–44, 46, 52, 63–73], in addition, medical records were used by others [22, 23, 40]. Twenty-six studies [6, 7, 9, 16–19, 21, 23, 26, 27, 40–43, 46, 52, 63–69, 71, 73] awarded ≥ 7 stars and remaining studies awarded 6 stars [5, 8, 20, 24, 44, 70] or 5stars [22, 25, 72], indicating the quality of included studies was generally good (Supplementary Table 4).

### ET and BC risk

Figure 4 summarized results of categorical and dose-response analyses based on association between ET and the BC risk. Twenty-three studies were eligible for categorical analyses on relationship of current ET use and BC risk, including 8,054 BC cases among current users. The summary RR of BC risk in current users versus non-users of ET was 1.14 (95% CI = 1.05–1.22) with substantial heterogeneity (*I^2^* = 78.2%, *P_heterogeneity_* < 0.001, *n* = 23). Five studies involving 1,017 BC incidences among former ET users were included, whereas no elevated risk (RR = 0.98, 95% CI = 0.91–1.05) without heterogeneity (*I****^2^*** = 1.8%, *P_heterogeneity_* = 0.4, *n* = 5) was explored among former user.

**Figure 2:**
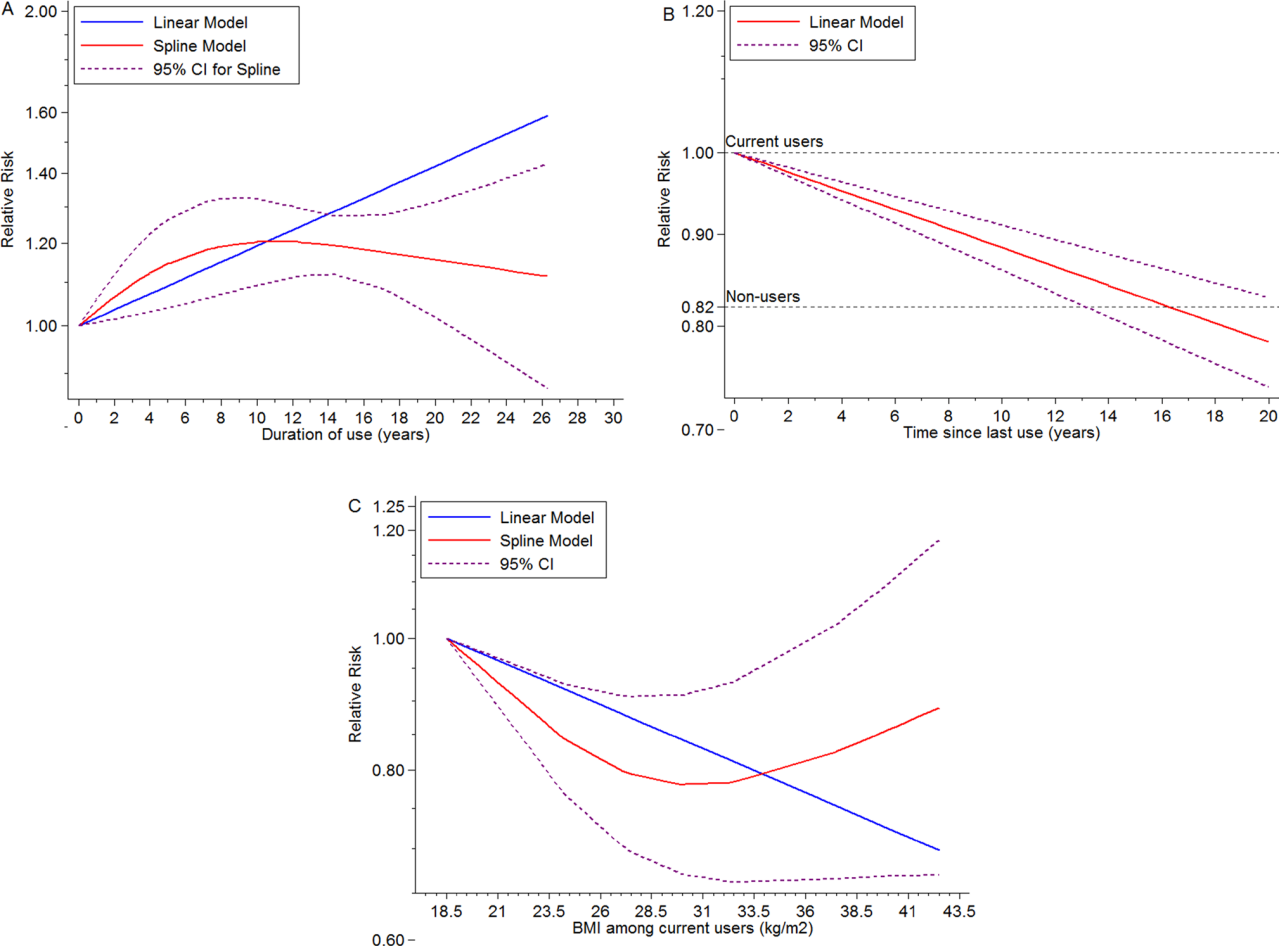
Dose-response meta-analyses on (**A**) duration of ET use (year) and the risk of BC compared with non-users, (**B**) time (year) since last ET use and the risk of BC compared with current ET users, (**C**) BMI (kg/m2) and the risk of BC among current ET users.

The random-effect dose-response on account of generalized least squares trend estimation showed the RR per year increase on duration of EP use was 1.02 (95% CI = 1.02–1.02, goodness-of-fit *chi*^2^_*42*_ = 114.79, *P_goodness-of-fit_*< 0.001, *n* = 15). In addition, we also explored a non-linear association between duration of ET use and BC risk (*P_non-linearity_* = 0.1) (Figure 2A) using restricted cubic spline model. The curve rose steeply and approximately reached the maximal RR at duration of 10 years, and declined slowly thereafter. Compared with current ET users, the dose-response association of time since last ET use and BC risk was found (RR for per year increase since last use = 0.99, 95% CI = 0.98–0.99, goodness-of-fit *chi^2^_18_* = 54.17, *P_goodness-of-fit_*< 0.001, *n* = 6), and a linear-relationship (*P_non-linearity_* = 0.45) (Figure 2B) indicated BC risk remained after going of ET regimen (RR for non-users compared with current users = 0.82, 95% CI = 0.71–0.93, *I^2^* = 63.5%, *P_heterogeneity_* = 0.02, *n* = 6) (Figure 4B). Additionally, among ET users, we found evidence of dose-response association between individual BMI and BC risk (per 5 units in BMI profile for RR = 0.93, 95% CI = 0.91–0.95, goodness-of-fit *chi^2^_17_* = 79.42, *P_goodness-of-fit_*< 0.001, *n* = 8). An “U” shaped (*P_non-linearity_* = 0.001) (Figure 2C) relationship of BMI with BC risk was documented among ever ET users, and the minimum risk approximately rested on the level of 30 kg/m^2^. Additionally, subgroup analyses, sensitivity analyses and publication biases were depicted in Table.1 and Supplementary Outcomes.

### EPT and BC risk

Table.2 summarized results of categorical and dose-response analyses based on association between EPT use and BC risk. Twenty-two studies were included in categorical analyses on relationship between EPT use and BC risk, including 17,584 cases among current EPT users. Compared with non-users, the summary RRs were 1.76 (95%CI = 1.56–1.96, n = 22) for current EPT use and 1.06 (95%CI = 0.93–1.20, n = 5) for former users. Substantial heterogeneity was observed between studies of current EPT users (*I^2^* = 92%, *P_heterogeneity_* < 0.001), but not among former users (*I^2^* = 48.3%, *P_heterogeneity_* = 0.1).

**Figure 3:**
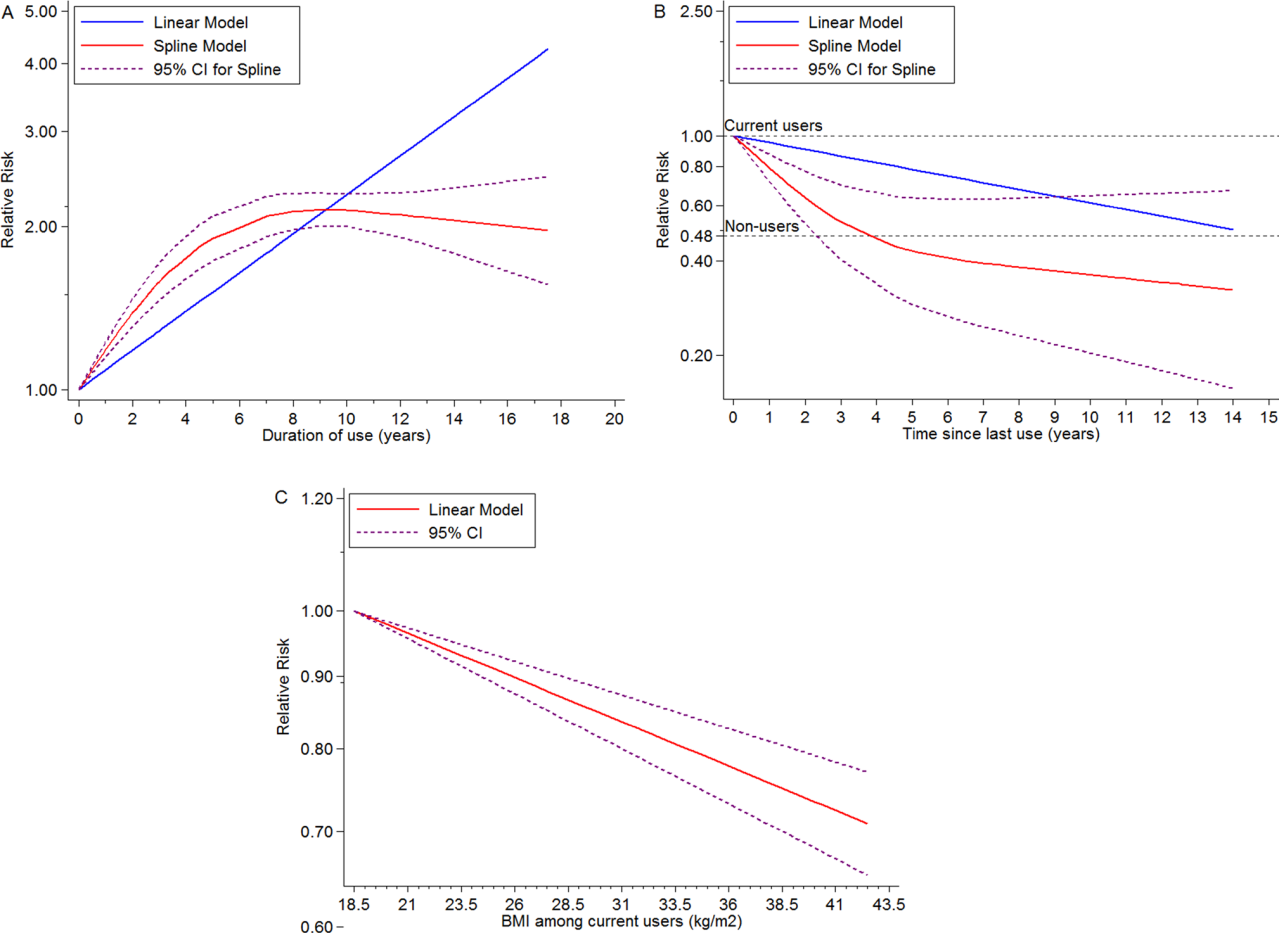
Dose-response meta-analyses on (**A**) duration of EPT use (year) and the risk of BC compared with non-users, (**B**) time (year) since last ET use and the risk of BC compared with current ET users, (**C**) BMI (kg/m2) and the risk of BC among current ET users.

There was significant evidence of dose-response association between duration of EPT use and the risk of BC, and summary RR for per year increase was 1.08 (95% CI = 1.08–1.08, goodness-of-fit *chi^2^_35_* = 296.40, *P_goodness-of-fit_* < 0.001, *n* = 14). At the same time, we found a non-linear association (*P_non-linearity_* < 0.001) (Figure 3A) between use time and BC risk, suggesting that an increasing trend displayed in first 5-year use and became gentle after that. Compared with current users, does-response relationship was detected according to time from EPT cessation and BC risk, indicating a dose-response association between per year increase in time since last EPT use and the risk of BC (RR for per year increase since last use = 0.95, 95% CI = 0.94–0.96, goodness-of-fit *chi^2^_16_* = 131.10, *P_goodness-of-fit_* < 0.0001, RR for non-users = 0.48, 95% CI = 0.28–0.69, *I^2^* = 98.1%, *P_heterogeneity_* < 0.001, n = 6), and a non-linear curve was revealed (*P_non-linearity_* < 0.0001) (Figure 3B). This spline indicated no EPT-associated BC risk remained after 4 years’ cessation, and potential benefit was observed. Linear dose-response relationship between BMI and the risk of BC among EPT users was found (RR for per 5 units increase in BMI = 0.93, 95% CI = 0.91–0.95, goodness-of-fit ***chi^2^***_*17*_ = 237.69, *P_goodness-of-fit_*< 0.001, *n* = 8) (*P_non-linearity_* = 0.37) (Figure 3C). Subgroup analyses, sensitivity analyses and publication biases were depicted in Figure 5 and Supplementary Outcomes.

**Figure 4:**
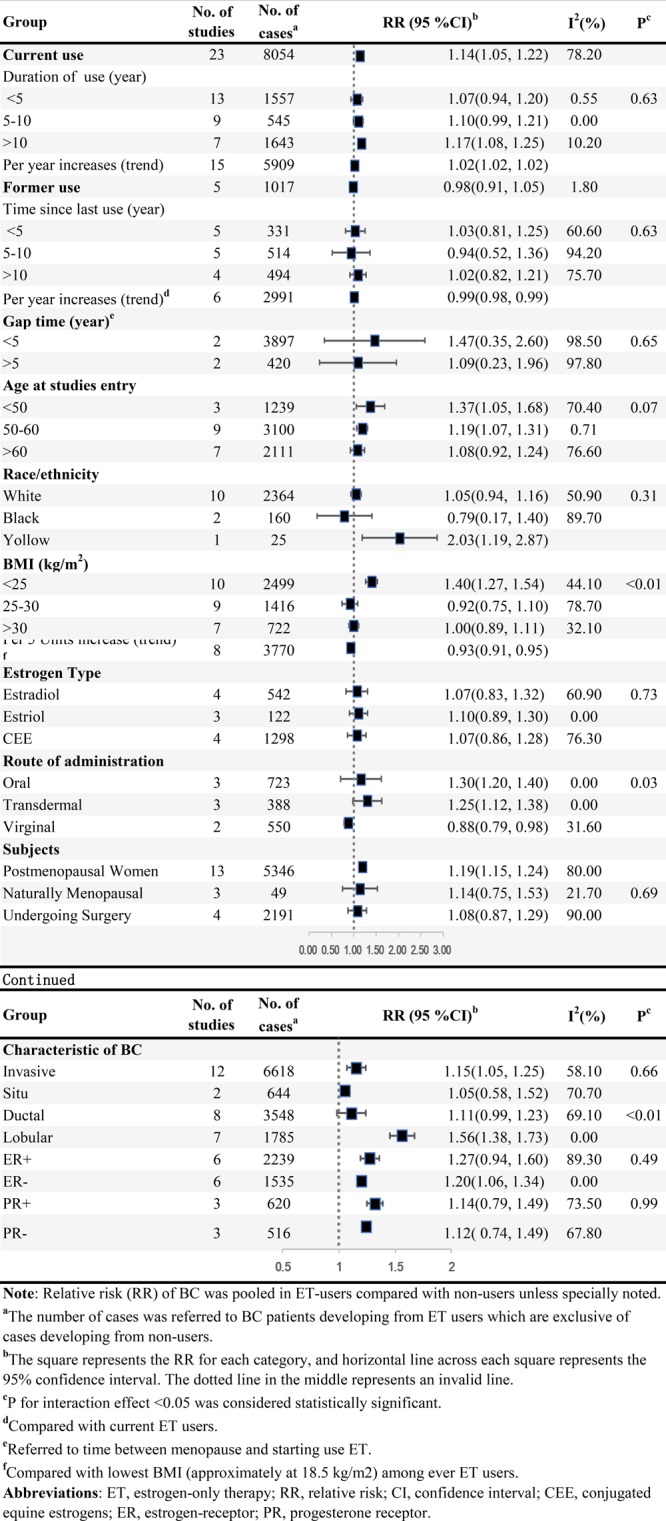
ET and the risk of BC

**Figure 5:**
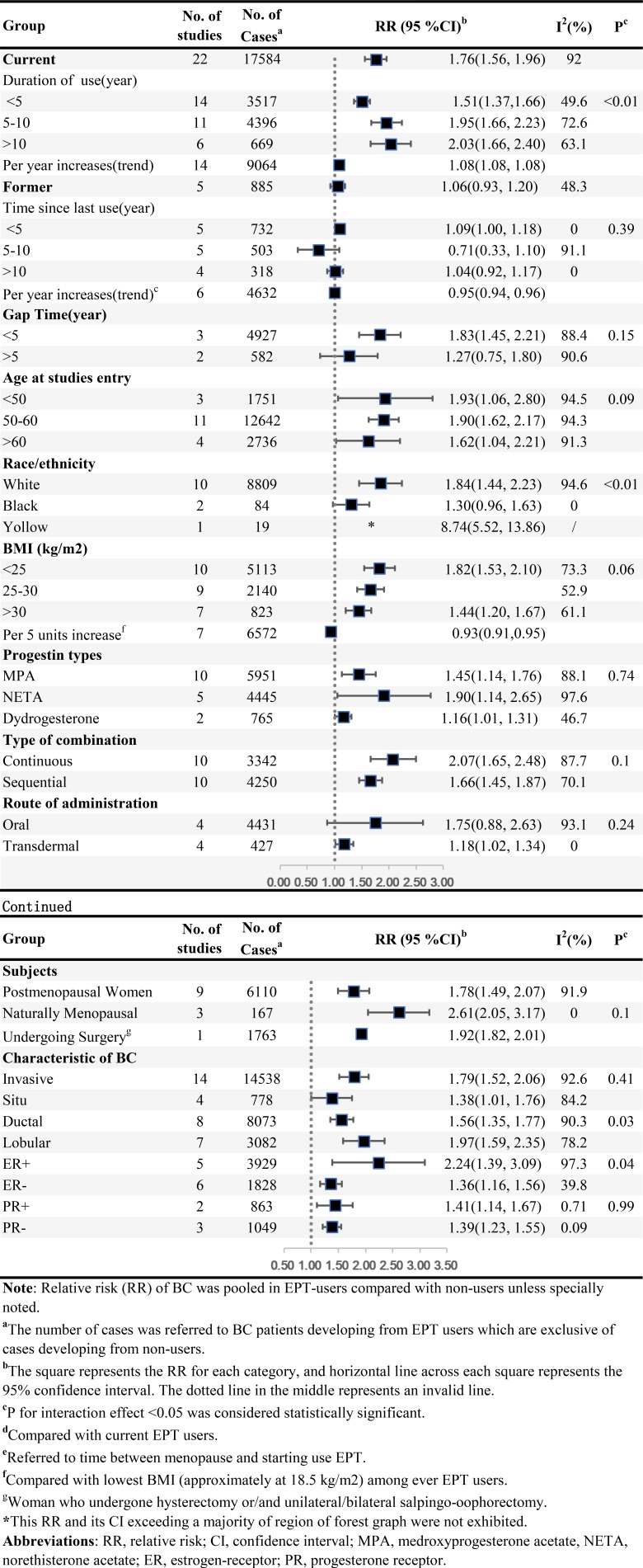
EPT and the risk of BC

### Other HRT regimens and BC risk

Supplementary Table 5 summarized results of categorical and dose-response analyses based on association between Other HRT regimens and BC risk. Either mixed HRT regimens or tibolone was associated with increased BC risk (RR for mixed HRT regimens = 1.50, 95% CI = 1.33–1.67, I^2^ = 91.3%, *P_heterogeneity_* < 0.001, n = 20; RR for per year increase in duration of mixed HRT regimens use = 1.04, 95% CI = 1.03–1.05, goodness-of-fit *chi2_35_* = 111.47, *P_goodness-of-fit_*< 0.001, n = 10 (Supplementary Figure 4); RR for tibolone = 1.47, 95% CI = 1.20–1.75, *I^2^* = 91.3%, *P_heterogeneity_* = 0.01, n = 5), whereas no association between PT use and the risk of BC was found (RR = 1.15, 95% CI = 0.86–1.44, *I^2^* = 16.4%, *P_heterogeneity_* = 0.31, *n* = 20).

## DISCUSSION

The present systematic review and meta-analysis of prospective studies reveals that HRT use is consistently associated with the elevated BC risk, regardless of ET, EPT as well as tibolone, whereas they show inconsistent magnitudes according to different effect-modifiers. Furthermore, the obviously disappeared or attenuated BC risks were observed among former HRT users, overweight or obesity users (BMI > 25kg/m^2^), long duration between menopausal and starting taking medications (> 5years), and somewhat benefit for BC prevention among former EPT users after ceasing 4 years was observed. Meanwhile, these inverse findings were further confirmed by the robust dose-response or its subgroup analyses. Other potential modifiers such as duration of use, route of administration and race/ethnicity were identified by subgroup analyses. The evidence of significant differences in characteristics of HRT-specific BC were found. Both ET and EPT are associated with higher risk of lobular BC than ductal BC, while more ER-positive BC cases than ER-negative BC were detected among EPT users.

Current thinking suggested that the level of EPT-associated BC risk seems to be greater than that of ET [74]. Either endogenous or exogenous of estrogen levels were generally considered as a causal role in the etiology of BC [75, 76], and a proposed hypothesis deeming that the carcinogenic effect of estrogens was augmented by progesterone [77, 78] explained disparity of two regimens, which is consistent with our results in regard to no association between PT and the risk of BC. Several robust mechanisms were addressed for two components based on this hypothesis via vivo or vitro studies [79, 80]. The carcinogenic effect of estrogen owned to increasing the mitotic rate of malignant or normal breast epithelium cells or combining its metabolites to exert effect [79, 80], contrarily, the progesterone indirectly realized synergies [81]. Additionally, we observed more risk of BC in subjects starting to use EPT or ET regimens less than 5 years after menopause, but no statistically significant BC risk was obtained when gap time > 5 years. HRT users with a long time from menopause to first HRT exposure exactly validated a plausible mechanism proposed as an estrogen-induced apoptosis model by previous estrogen deprivation. [82].

In respect to duration of HRT regimens use, similar “inverted-U” non-linear relationships of either ET or EPT regimen with the risk of BC were observed. Consistent curves were also documented among the Breakthrough Generations Study (GBS) [6], the Breast Cancer Detection Demonstration Project Study(BCDDPS) [67] and the National Swedish Cancer Registry study(NSCRS) [64], indicating the onset peaks are proximately at 8–10 years after persistently EPT, ET using, respectively. This BC incidence peak is interpretable, for the opinion regarding a promotional effect of HRT regimens on occult tumors was come up with [83]. Several autopsy studies suggested that this reservoir of small, occult, undiagnosed BC is so common up to 15.6 percent among dying women from unrelated causes with more than 10 years growth until clinical detecting [83], and this time was shorten notably by EPT regimens [83]. As subgroup analysis regarding age at studies entry shown, the elderly users owned the relatively lower BC risk than mid-aged. The subsequent decline of curve was possibly explained by growing age and biases from inadequate adherence or limited sample size for long-time users, in addition of that, restricted numbers of studies with duration of use > 10 years contributing to considerable biases. Moreover, some non-users may start using HRT after long time, and we often observed smaller risk gap between “non-users” and long-term users than initial groups [84].

Interestingly, disparity of residual effects between ET and EPT on BC risk after stopping drugs was first revealed by this study. Although ET was found to be safer than EPT in regard to BC incident, the minor BC risk remained after drug withdrawal, which arose widespread concerns in previous studies [6, 9, 26, 40, 67]. Jones et al observed consistent results with us, compared with no previous HRT use, 2.1 percent and -1 percent increase in HR per year since last use among past users among participants receiving ET and EPT, respectively [6]. Moreover, differences tended to be obvious when stratified analyses were conducted according to duration of former HRT use, and participants with more than 5-year HRT use showed 2.4 and 0.88 magnitudes of ET and EPT related residual BC risk compared with non-users, respectively [9]. We failed to investigate the potential influences from duration of former HRT use due to limited evidence of the respect. The persistent residual BC risk made clinicians cautious when prescribing ET regimens, and more well-designed studies with long follow up was urgently needed to explore the effect of cessation of HRT use on BC risk.

Efforts to interpret inconsistent outcomes between RCT and observational studies suggested that BMI is an important modulator of HRT's influence on BC risk [6, 9, 13, 16, 18, 20, 21, 26, 27, 67]. As interesting as all this is, among several large-scale observational studies, average BMI of participants was about 25kg/m^2^ (e.g. 24.8 kg/m^2^ (the European Prospective Investigation into Cancer and Nutrition [16]); 26.2 kg/m^2^ (the Million Women Study [27]); 25.7 kg/m^2^ (the Breakthrough Generations Study (GBS) [6]); 24.9 kg/m^2^ (the Nurses’ Health Study [19])), which is notably lower than that in WHI trial (28.5 kg/m^2^) that is the minimum risk point of our “U” shaped of our curve. Thus, BMI disparity between observational studies and the RCT have possibility to lead underestimation of HRT-associated BC risk reported by WHI RCT. The random-effect dose-response on account of generalized least squares trend estimation explored a reduction in BC incidence with the increase of BMI among HRT users, consistent results had been found in previous studies [15, 17, 19, 27]. Furthermore, non-users with higher BMI are often associated with elevated risk of BC, which was validated by a comprehensive dose-response meta-analysis [85], indicating that smallest BMI-associated ‘risk gap’ in BC between non-exposure and exposure existed in WHI, which is likely to lead no statistically significant magnitude of ET-related BC risk.

Combinations and administrations for HRT were critical modifiers, which were recognized in previous studies [5, 9, 22, 23, 25, 27, 63, 86]. Most studies suggested that BC risk was greater with continuous-combined than sequential regimen [5, 18, 22, 26, 41, 52], and the results were consistent with us. Oral progestin in sequential regimen can efficiently counteract the hyperplastic effect of estrogen [87], but no differences of BC risk between oral and transdermal EPT use were identified. Inversely, ET particularly orally administered estrogens are considered as counteract metabolic factors increasing the risk of BC, which were achieved by increasing insulin sensitivity and lowering circulating insulin levels [86], and this study suggested similar results that oral-ET had highest BC risk than transdermal and virginal use.

The notable strength of the dose-response meta-analysis with systematic review was to clarify the associations between HRT use and the risk of BC and related modifiers, and to the best of our knowledge, we are the first to explore dose-response relationships of changing year-to-year for HRT use or withdrawal and BMI among HRT users with BC risk. Nevertheless, some limitations of our study should be acknowledged. Firstly, considering that our meta-analysis was based on study-level, our results therefore exhibited a lack of reliability relative to previous individual patient data meta-analysis [15]. Most data were extracted from outcomes of primary analyses, but others such as age and race/ethnicity were roughly extracted from characteristics of sample population, which therefore contributed to failure of draw conclusions on subgroup analyses of them. Secondly, we failed to investigate the influences of mammary screening and breast density on BC risk in subjects using HRT duo to limited related studies. Lastly, stratified analyses were conducted in our study, and most eligible studies adjusted related variables, identifying several key factors such as duration of HRT use or quit, BMI and gap time. However, high heterogeneity in this study was documented coming from clinical and methodological aspects, and unmeasured or residual modifiers can't be eliminated.

## CONCLUSIONS

This study supports that current use of ET, EPT and ever use of tibolone increased the risk of BC, whereas no association of former ET, EPT use or ever PT use with BC risk is identified. For obvious heterogeneity among included studies is presented, these outcomes should be interpreted with caution. We also further summary current epidemiological evidences according to sever proposed interactions, suggesting that duration of HRT use, time since last HRT use, gap time and BMI are potential modifiers. HRT users regardless of ET or EPT with longer duration of use are associated with higher BC risk with 8–10 years’ onset peaks, but longer time from last HRT use, longer gap time and higher BMI contribute to attenuated BC risk. Both regimens are associated with higher risk of lobular BC than ductal BC, and more ER-positive BC cases than ER-negative BC were detected among EPT users. More studies with larger sample size and better control of these effect-modifiers will be needed to reveal dose-response effects on HRT regimens or their modifiers and the risk of BC.

## SUPPLEMENTARY MATERIALS FIGURES AND TABLES







## Abbreviations

HRT: hormone replacement therapy
ET: estrogen-alone therapy
EPT: estrogen plus progestin therapy
BC: breast cancer
WHI: women health initial
pRCT: randomized clinical trial
RR: relative risk
CI: confidence interval
NOS: Newcastle-Ottawa quality assessment scale
ER: estrogen receptor
PR: progesterone receptor
PT: progestin-only therapy
MPA: medroxyprogesterone acetate
NETA: norethisterone acetate
BMI: body mass index
CEE: conjugated equine estrogens.
