# Supplementary material for: Change in risk of breast cancer after receiving hormone replacement therapy by considering effect-modifiers: a systematic review and dose-response meta-analysis of prospective studies
Source: Oncotarget. 2017 Aug 11;8(46):81109–24. doi: 10.18632/oncotarget.20154 (PMC5655266; doi:10.18632/oncotarget.20154)
Supplement: Supplementary file 1 [file oncotarget-08-81109-s001.pdf]

Change in risk of breast cancer after receiving hormone replacement therapy by considering effect-modifiers: a systematic review and dose-response meta-analysis of prospective studies

SUPPLEMENTARY MATERIALS

ET and the risk of BC

In subgroup analyses ET on BC risk, no modification effect and difference showed on duration of ET use, time since last use, race/ethnicity, estrogen type, subjects and expression of ER and PR, invasive or not (all $P_{interaction} > 0.05$). Nevertheless, the statistically elevated risk of BC was observed when duration of ET use extended to more than 10 years, compared with duration in less than 10 years. The BC RRs for participants at < 50, 50–60, > 60 years at studies entry were respectively 1.37 (95% CI = 1.05–1.68, I^2 = 70.4%, $P_{heterogeneity}$ = 0.03, n = 3), 1.19 (95% CI = 1.07–1.31, I^2 = 70.6%, $P_{heterogeneity}$ < 0.01, n = 9), 1.08(95% CI = 0.92–1.24, I^2 = 70.6%, $P_{heterogeneity}$ < 0.001, n = 7), and marginal interaction effect was found ($P_{interaction}$= 0.07). Meanwhile, the risk of BC among ET users varied substantially ($P_{interaction}$< 0.01) according to BMI of subjects. There was moderate increased risk (RR = 1.40, 95% CI = 1.27–1.54, n = 9) at low and normal BMI rang down to a BMI of 25kg/m^2, but risk disappeared in the overweight (BMI of 25-30 kg/m^2) and obesity (BMI of > 30 kg/m^2) categories. On the basis of limited studies, different routes of administration potentially affected BC risk ($P_{interaction}$= 0.02), one of which exerted a protectable effect on BC incidence when women using ET vaginally (RR=0.88, 95% CI = 0.79–0.98, n = 2). Furthermore, this association was considerably stronger among postmenopausal women (RR=1.19, 95% CI = 1.15–1.24, n = 13), but failed to find significant associations when restricting women to those undergoing surgeries or naturally menopause. As for characteristics of BC, BC risk differed between ductal (RR= 1.11, 95% CI = 0.99–1.23, I^2 = 69.1%, $P_{heterogeneity}$ < 0.01, n = 8) and lobular (RR = 1.56, 95% CI=1.38–1.73, I^2 = 0, $P_{heterogeneity}$ = 0.69, n = 7) carcinoma($P_{interaction}$< 0.01). When restricting dose-response meta-analysis for participants with BMI < 25 kg/m^2, there was no dose-response relationship between duration of use and BC risk (RR for per year increase in duration of ET use = 1.03, 95% CI = 1.02–1.03, goodness-of-fit chi^2_{18} = 27.85, $P_{goodness\text{-}of\text{-}fit}$ = 0.06, n =6), and a linear curve on this relationship was found ($P_{non\text{-}linearity}$ = 0.12) (eFigure 1A in the Supplementary). Similarly, no dose-response associations between duration of ET use and the risk of BC among overweight and obesity participants (BMI > 25 kg/m^2) was reveled through a linear random-effect dose-response model (RR for per year increase in duration of use=1.01, 95% CI = 1.00–1.01, goodness-of-fit chi^2_{18} = 15.58, $P_{goodness\text{-}of\text{-}fit}$ = 0.62, n = 6), and a linear curve was detected ($P_{non\text{-}linearity}$ =0.53) (eFigure.1B in the Supplementary). Based on association of current ET use and the risk of BC, omitting one study in turn did not significantly change the summary risk estimate of BC, with the pooled RRs ranging from 1.14 (95% CI = 1.07 to 1.22)[1] to 1.18 (95% CI = 1.10 to 1.27)[2]. Furthermore, no evidence of publication bias for this relationship was found by Begg's test and Egger's test (all P > 0.05).

EPT and the risk of BC

It seems that only duration of EPT use, race/ethnicity and type of combined regimen can explain the high heterogeneity for relationship of EPT with the increased BC risk (all $P_{interaction}$< 0.05). The summary RRs showed stronger with long time use (> 10 years), ER positive or lobular BC (RR= 2.03, 1.97, 2.24, 95% CI=1.66–2.40, 1.59–2.35, 1.39–3.09, respectively). Remarkably, some variables like BMI, age at entry and type of combined regimen were critically potential modifiers (all $P_{interaction}$< 0.1), and a significant higher BC risk was observed in a thinner and younger woman using continuous EPT regimens. When further investigating modification effect of BMI or gap time on dose-response analyses for duration of use, similar magnitudes were observed among low BMI (< 25 kg/m^2) (RR for per year increase in duration of use = 1.06, 95% CI = 1.05–1.06, goodness-of-fit chi^2_{12}= 61.10, $P_{goodness\text{-}of\text{-}fit}$< 0.001, n = 5) ($P_{non\text{-}linearity}$ = 0.001) (eFigure 2A in the Supplementary) and high BMI (> 25 kg/m^2) participants (RR for per year increase in duration of use=1.05, 95% CI = 1.04–1.06, goodness-of-fit chi^2_{12}=28.42, $P_{goodness\text{-}of\text{-}fit}$< 0.01, n = 5) ($P_{non\text{-}linearity}$ =0.49) (eFigure 2B in the Supplementary). Additionally, we identified that dose-response curve for the relationship of duration of EPT use with BC risk was linear in users with gap time < 5 years (RR for per year increase in duration of use = 1.11, 95% CI = 1.10–1.11, goodness-of-fit chi^2_{12} = 198.76, $P_{goodness\text{-}of\text{-}fit}$< 0.001, n = 3) ($P_{non\text{-}linearity}$ = 0.57) (eFigure.3A in the Supplementary) and in those with gap time > 5 years (RR for per year increase in duration of use = 1.07, 95% CI =

1.05–1.09, goodness-of-fit chi^2_{12} = 14.38, $P_{\text{goodness-of-fit}}$ = 0.01, n = 2) ($P_{\text{non-linearity}}$ =0.41) (eFigure3B in the Supplementary). Regarding to association between current EPT use and the risk of BC, each study omitting in turn did not alter the initial outcome, with magnitude changing from 1.75 (95% CI = 1.57 to 1.96)[3] to 1.88 (95% CI = 1.68 to 2.11)[4]. Meanwhile, no small study bias was identified by Begg's test and Egger's test (all P > 0.05).

Other HRT regimens and BC risk

Either mixed HRT regimens or tibolone was associated with increased BC risk (RR for mixed HRT regimens = 1.50, 95% CI = 1.33–1.67, I^2 = 91.3%, $P_{\text{heterogeneity}}$ < 0.001, n = 20; RR for per year increase in duration of mixed HRT regimens use = 1.04, 95% CI = 1.03–1.05, goodness-of-fit chi^2_{35} = 111.47, $P_{\text{goodness-of-fit}}$<0.001, n=10 (*eFigure4 in the Supplement*); RR for tibolone = 1.47, 95% CI = 1.20–1.75, I^2 = 91.3%, $P_{\text{heterogeneity}}$ =0.01, n = 5), whereas no association between PT use and the risk of BC was found (RR = 1.15, 95% CI = 0.86–1.44, I^2 = 16.4%, $P_{\text{heterogeneity}}$ =0.31, n = 20)t

Supplementary Table 1: Search history of pubmed search strategy

No.	Search strategy	Items
#1	"Breast"[Mesh]	39,618
#2	(Breast[Title/Abstract]) OR Mammary[Title/Abstract]	384,186
#3	#1 OR #2	390,782
#4	"Neoplasms"[Mesh]	2,814,649
#5	((((Cancer[Title/Abstract]) OR Neoplasm[Title/Abstract]) OR Tumors[Title/Abstract]) OR Malignancy[Title/Abstract]) OR Carcinoma[Title/Abstract]	1,931,285
#6	#4 OR #5	3,238,428
#7	"Hormone Replacement Therapy"[Mesh]	22,380
#8	((((((((((Hormone replacement therapy[Title/Abstract]) OR menopausal hormone therapy[Title/Abstract]) OR estrogen therapy[Title/Abstract]) OR estrogen and progestin therapy[Title/Abstract]) OR tibolone[Title/Abstract]) OR progestin [Title/Abstract]) OR medroxyprogesterone acetate [Title/Abstract]) OR norethisterone acetate [Title/Abstract]) OR dydrogesterone [Title/Abstract]) OR estradiol [Title/Abstract]) OR estriol [Title/Abstract]) OR conjugated equine estrogens [Title/Abstract]	897,682
#9	#7 OR #8	102,322
#10	#3 AND #6 AND #9	4,055
#11	review [Publication Type] OR letter [Publication Type]	3,064,769
#12	#10 NOT #11 Filters: Humans	2,350

PubMed search strategy with superior search capabilities was adapted for use with the other databases.
Items were found from EMBASE database (n = 2071) and Web of Science database (n = 1325) until Jan 3, 2017.

Supplementary Table 2: Reasons for exclusion of full manuscripts screened and not included in meta-analysi. See Supplementary_Table_2

Supplementary Table 3: Characteristics of included studies. See Supplementary_Table_3

Supplementary Table 4: Results of quality assessment for thirty-five unique studies based on the Newcastle-Ottawa quality assessment scale (NOS)

Source (reference)	Selection11				Comparability[2]		Outcome[3]			Total Score[4]
	Representativeness of exposed cohort[5] ☆	Selection of non-exposed cohort[6] ☆	Exposure ascertainment[7] ☆	No cases when investigatio -ns begin ☆	Comparable on confounder8 ☆ ☆		Outcome Assessment[9] ☆	Adequate follow-up (≧5y) ☆	Loss to follow-up rate (≧20%) ☆	
Roman et al.[5]	☆	☆		☆	☆		☆		☆	6
Jones et al.[6]	☆	☆	☆	☆	☆		☆	☆	☆	8
Suhrke et al.[7]	☆	☆	☆	☆	☆		☆	☆	☆	8
Chlebowski et al.[2]	☆	☆	☆	☆		☆	☆	☆		7
Thorbjarnardo-ttir et al.[15]	☆	☆	☆	☆	☆			☆		6
Fournier et al.[16]		☆	☆	☆	☆	☆	☆	☆	☆	8
Ritte et al.[19]	☆	☆	☆	☆	☆	☆	☆	☆	☆	9
Lai et al.[3]	☆	☆	☆	☆			☆	☆	☆	7
Beral et al.[1]	☆	☆	☆	☆	☆	☆	☆		☆	8
Saxena et al.[22]		☆	☆	☆	☆	☆	☆	☆		7
Kotsopoulos et al.[23]		☆	☆	☆	☆		☆	☆	☆	7
Lyytinen et al.[25]	☆	☆		☆			☆	☆		5
Calle et al.[26]	☆	☆	☆	☆	☆	☆	☆	☆	☆	9
Brinton et al.[27]	☆	☆	☆	☆	☆		☆	☆		7
Espiéet et al.[28]	☆	☆	☆	☆				☆		5
Rosenberg et al.[29]	☆	☆	☆	☆	☆		☆	☆	☆	8
Ewertz et al.[4]	☆	☆	☆	☆	☆	☆	☆	☆	☆	9
Tjonneland et al.[30]	☆	☆	☆	☆	☆		☆			6
Stahlberg et al.[31]		☆	☆	☆	☆		☆	☆		6
Feigelson et al.[32]	☆	☆	☆	☆	☆	☆	☆	☆	☆	9
Bakken et al.[33]	☆	☆	☆	☆	☆	☆		☆		7
Olsson et al.[34]	☆	☆	☆	☆	☆		☆	☆	☆	8
Jernstrom et al.[35]	☆	☆	☆	☆	☆		☆		☆	7
Lignieres et al.[36]	☆	☆	☆	☆	☆		☆	☆	☆	8
Chen et al.[37]	☆	☆	☆	☆	☆		☆	☆	☆	8
Manjer et al.[38]	☆	☆	☆	☆	☆	☆	☆	☆	☆	9
Schairer et al.[39]	☆	☆	☆	☆	☆	☆	☆	☆		8
Persson et al.[40]	☆	☆	☆	☆	☆	☆	☆	☆	☆	9
Lando et al.[41]	☆	☆	☆	☆	☆	☆		☆	☆	8
Gapstur et al.[42]	☆	☆	☆	☆	☆	☆	☆	☆	☆	9
Sourander et al.[43]	☆	☆	☆	☆			☆	☆	☆	7
Schuurman al[44]	☆	☆	☆	☆	☆	☆	☆		☆	8
Risch et al.[45]	☆	☆	☆	☆				☆		5
Mills et al.[46]	☆	☆	☆	☆			☆	☆		6
Adami et al.[47]	☆	☆	☆	☆	☆	☆	☆	☆		8

[1] "Selection" part includes representativeness of cases, selection of controls, exposure ascertainment, and no breast cancer incident when investigation begin.
[2] "Comparability" part includes comparable on confounders.
[3] "Outcome" part includes outcome assessment, adequate follow-up, and loss to follow-up rate.
[4] The total score is equal to the total number of stars.
[5] The exposed cohorts are population-based or hospital-based, which can award a star. Population coming from nurses, volunteers or unknown sources cannot award a star.
[6] Only the same source with exposed cohorts, the non-exposed cohorts are considered as selected.
[7] When exposure variables should be ascertained by medical records or questionnaires, a star should be awarded.
[8] If the studies adjusted for two individual factors among age, menopausal age, family history of breast cancer, mammographic screening, it is award a star. If the studies adjusted for two lifestyle factors among body mass index, physical activity, smoking status and alcohol consumption, it is award another star.
[9] When breast cancer cases were identified by cancer registry, pathological proving or medical records, a star should be awarded.

Supplementary Table 5: Other HRT regimens and the risk of BC

Group	No. of studies	No. of Cases[a]	RR (95 %CI)[b]	I^2(%)	$P_{heterogeniety}$	$P_{interaction}$
**Mixed HRT regimens**	19	11026	1.52(1.36, 1.69)	86.7	<0.001	
**Duration of ever use (years)**						
<5	10	909	1.28(1.09, 1.48)	57.5	<0.01	0.5
5-10	8	372	1.46(1.03, 1.89)	82.7	<0.001	
>10	7	177	1.68(1.22, 2.15)	62.5	<0.01	
Increase in RR per year of use among ever users (trend)	10		1.04(1.03, 1.05)			
**Progestin-Only**	6	92	1.15(0.86, 1.44)	16.4	0.31	
**Tibolone**	5	727	1.47(1.20, 1.75)	71.1	0.01	

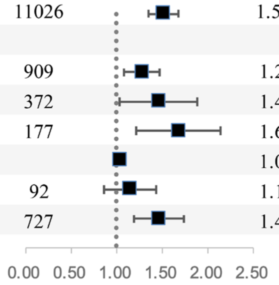

Note: Relative risk (RR) of BC was pooled in EPT-users compared with non-users.

[a]The number of cases was referred to BC patients developing from EPT users which are exclusive of cases developing from non-users.

[b]The square represents the RR for each category, and horizontal line across each square represents the 95% confidence interval. The dotted line in the middle represents an invalid line.

Abbreviations: RR, relative risk; CI, confidence interval; HRT, hormone replacement therapy.

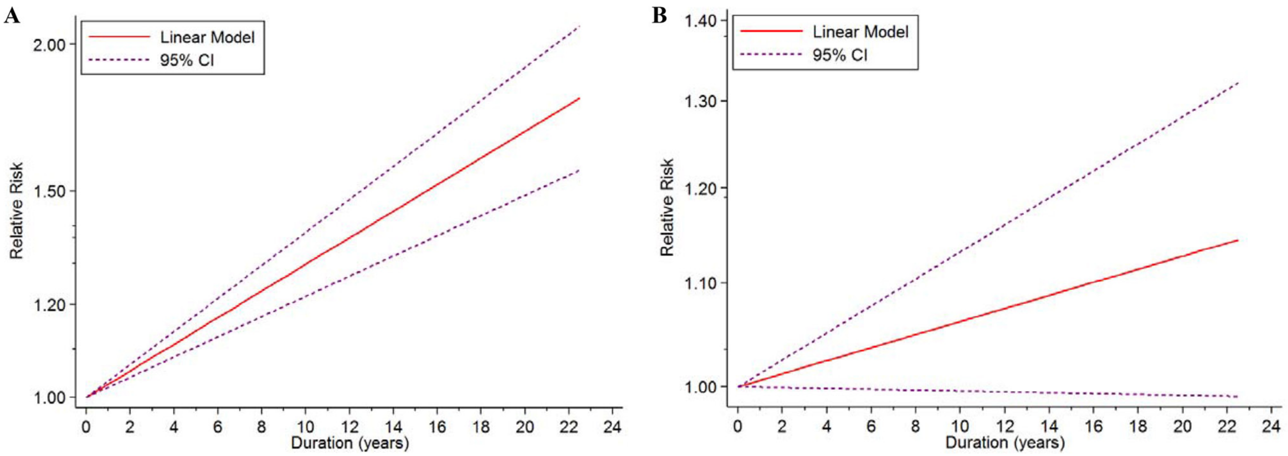

Supplementary Figure 1: Dose-response meta-analysis on duration of ET use (year) and the risk of BC among subjects with (**A**) BMI < 25 kg/m^2 and (**B**) BMI > 25 kg/m^2 compared with non-users, respectively.

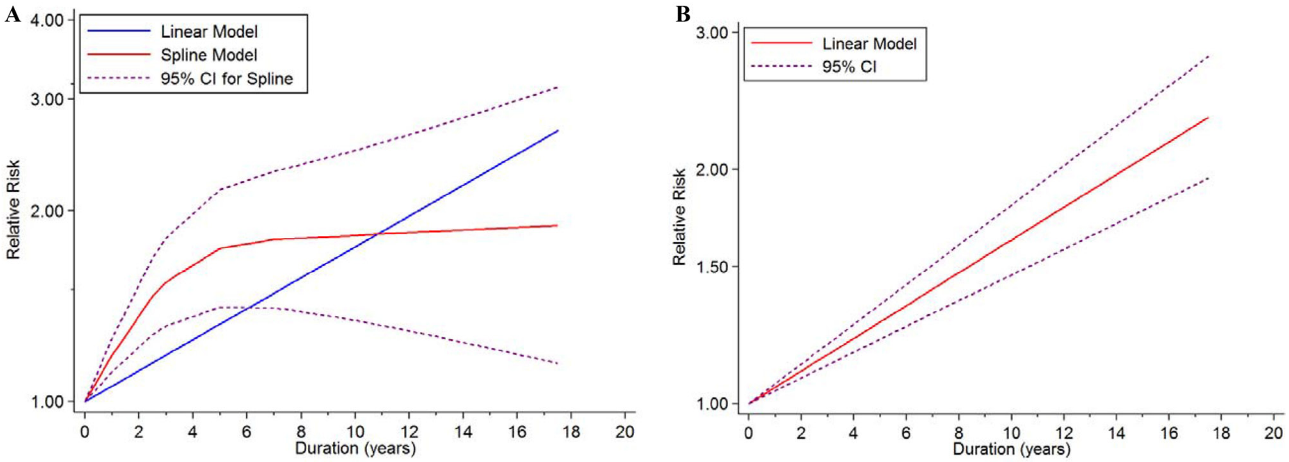

Supplementary Figure 2: Dose-response meta-analysis on duration of EPT use (year) and the risk of BC among subjects with (**A**) BMI < 25 kg/m^2 and (**B**) BMI > 25 kg/m^2 compared with non-users, respectively.

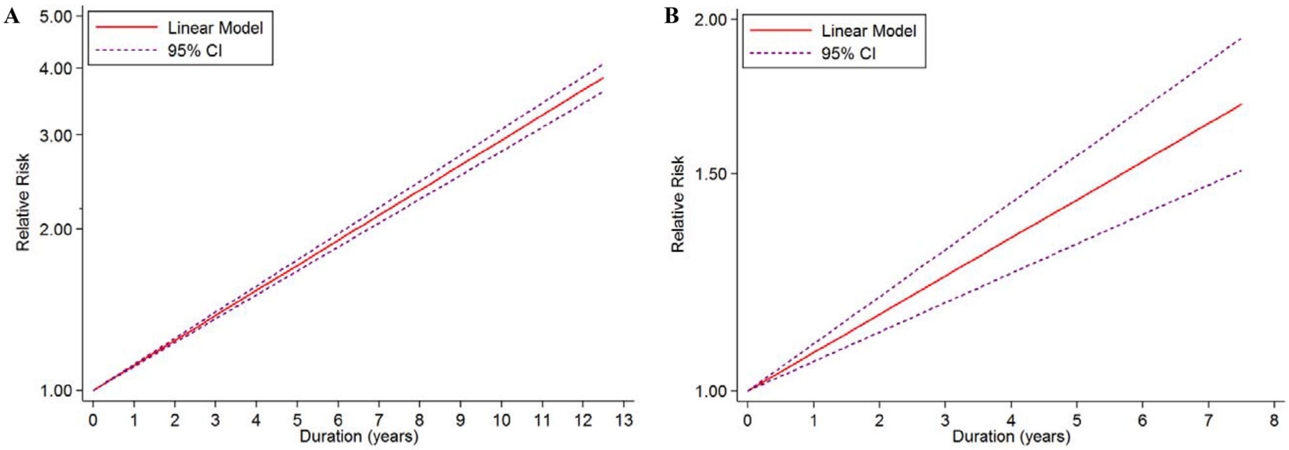

Supplementary Figure 3: Dose-response meta-analysis on duration of EPT use (year) and the risk of BC among users with (**A**) gap time < 5 years and (**B**) gap time > 5 years compared with non-users, respectively.

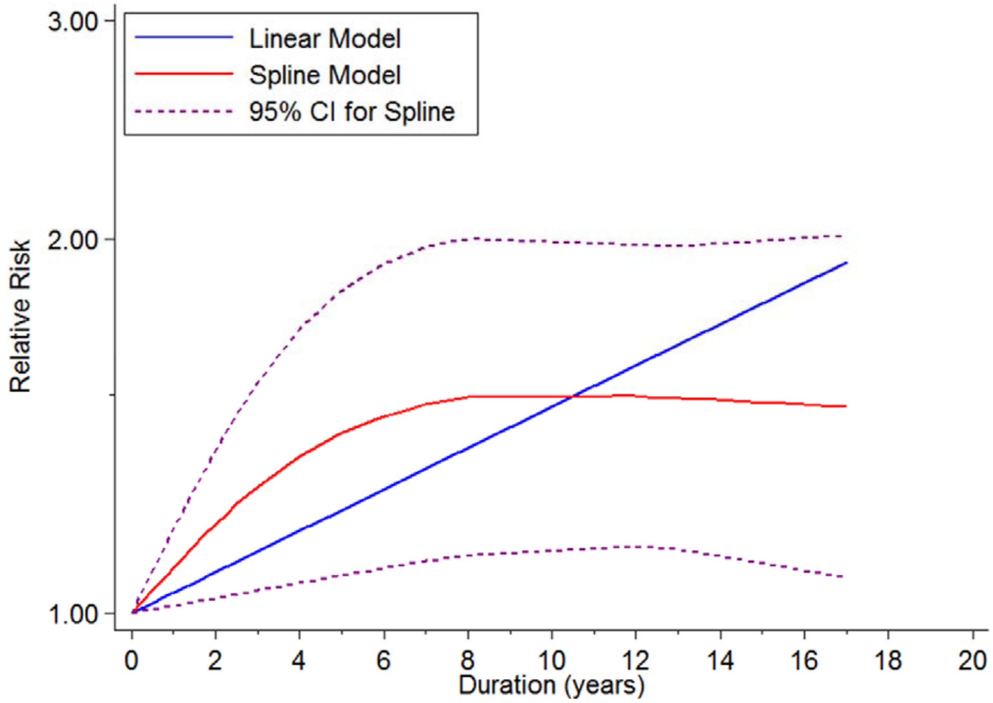

Supplementary Figure 4: Dose-response meta-analysis on duration of mixed HRT use (year) and the risk of BC.

REFERENCES

1. Beral V, Reeves G, Bull D, Green J, Million Women Study C. Breast Cancer Risk in Relation to the Interval Between Menopause and Starting Hormone Therapy. Journal Of the National Cancer Institute. 2011; 103:296–305. https://doi.org/10.1093/jnci/djq527.
2. Chlebowski RT, Rohan TE, Manson JE, Aragaki AK, Kaunitz A, Stefanick ML, Simon MS, Johnson KC, Wactawski-Wende J, O'Sullivan MJ, Adams-Campbell LL, Nassir R, Lessin LS, et al. Breast Cancer After Use of Estrogen Plus Progestin and Estrogen Alone: Analyses of Data From 2 Women's Health Initiative Randomized Clinical Trials. JAMA Oncol. 2015; 1:296–305. https://doi.org/10.1001/jamaoncol.2015.0494.
3. Lai JN, Wu CT, Chen PC, Huang CS, Chow SN, Wang JD. Increased risk for invasive breast cancer associated with hormonal therapy: a nation-wide random sample of 65,723 women followed from 1997 to 2008. PLoS One. 2011; 6:e25183. https://doi.org/10.1371/journal.pone.0025183.
4. Ewertz M, Mellemkjaer L, Poulsen AH, Friis S, Sorensen HT, Pedersen L, McLaughlin JK, Olsen JH. Hormone use for menopausal symptoms and risk of breast cancer. A Danish cohort study. Br J Cancer. 2005; 92:1293–7. https://doi.org/10.1038/sj.bjc.6602472.
5. Roman M, Sakshaug S, Graff-Iversen S, Vangen S, Weiderpass E, Ursin G, Hofvind S. Postmenopausal hormone therapy and the risk of breast cancer in Norway. Int J Cancer. 2016; 138:584–93. https://doi.org/10.1002/ijc.29810.
6. Jones ME, Schoemaker MJ, Wright L, McFadden E, Griffin J, Thomas D, Hemming J, Wright K, Ashworth A, Swerdlow AJ. Menopausal hormone therapy and breast cancer: what is the true size of the increased risk? Br J Cancer. 2016. https://doi.org/10.1038/bjc.2016.231.
7. Suhrke P, Zahl PH. Breast cancer incidence and menopausal hormone therapy in Norway from 2004 to 2009: A register-based cohort study. Cancer Medicine. 2015; 4:1303–8.
8. Rosenberg L, Bethea TN, Viscidi E, Hong CC, Troester MA, Bandera EV, Haiman CA, Kolonel LN, Olshan AF, Ambrosone CB, Palmer JR. Postmenopausal Female Hormone Use and Estrogen Receptor-Positive and -Negative Breast Cancer in African American Women. J Natl Cancer Inst. 2016; 108. https://doi.org/10.1093/jnci/djv361.

9. Luo JH, Cochrane BB, Wactawski-Wende J, Hunt JR, Ockene JK, Margolis KL. Effects of menopausal hormone therapy on ductal carcinoma in situ of the breast. Breast Cancer Research And Treatment. 2013; 137:915–25. https://doi.org/10.1007/s10549-012-2402-0.

10. Anderson GL, Chlebowski RT, Aragaki AK, Kuller LH, Manson JE, Gass M, Bluhm E, Connelly S, Hubbell FA, Lane D, Martin L, Ockene J, Rohan T, et al. Conjugated equine oestrogen and breast cancer incidence and mortality in postmenopausal women with hysterectomy: extended follow-up of the Women's Health Initiative randomised placebo-controlled trial. Lancet Oncology. 2012; 13:476–86. https://doi.org/10.1016/s1470-2045(12)70075-x.

11. LaCroix AZ, Chlebowski RT, Manson JE, Aragaki AK, Johnson KC, Martin L, Margolis KL, Stefanick ML, Brzyski R, Curb JD, Howard BV, Lewis CE, Wactawski-Wende J. Health outcomes after stopping conjugated equine estrogens among postmenopausal women with prior hysterectomy: a randomized controlled trial. JAMA. 2011; 305:1305–14. https://doi.org/10.1001/jama.2011.382.

12. Prentice RL, Chlebowski RT, Stefanick ML, Manson JE, Pettinger M, Hendrix SL, Hubbell FA, Kooperberg C, Kuller LH, Lane DS, McTiernan A, O'Sullivan MJ, Rossouw JE, et al. Estrogen plus progestin therapy and breast cancer in recently postmenopausal women. American Journal Of Epidemiology. 2008; 167:1207–16. https://doi.org/10.1093/aje/kwn044.

13. Anderson GL, Limacher M, Assaf AR, Bassford T, Beresford SA, Black H, Bonds D, Brunner R, Brzyski R, Caan B, Chlebowski R, Curb D, Gass M, et al. Effects of conjugated equine estrogen in postmenopausal women with hysterectomy: the Women's Health Initiative randomized controlled trial. JAMA. 2004; 291:1701–12. https://doi.org/10.1001/jama.291.14.1701.

14. Rossouw JE, Anderson GL, Prentice RL, LaCroix AZ, Kooperberg C, Stefanick ML, Jackson RD, Beresford SA, Howard BV, Johnson KC, Kotchen JM, Ockene J. Risks and benefits of estrogen plus progestin in healthy postmenopausal women: principal results From the Women's Health Initiative randomized controlled trial. JAMA. 2002; 288:321–33.

15. Thorbjarnardottir T, Olafsdottir EJ, Valdimarsdottir UA, Olafsson O, Tryggvadottir L. Oral contraceptives, hormone replacement therapy and breast cancer risk: A cohort study of 16 928 women 48 years and older. Acta Oncologica. 2014; 53:752–8.

16. Fournier A, Mesrine S, Dossus L, Boutron-Ruault MC, Clavel-Chapelon F, Chabbert-Buffet N. Risk of breast cancer after stopping menopausal hormone therapy in the E3N cohort. Breast Cancer Research and Treatment. 2014; 145:535–43.

17. Fournier A, Mesrine S, Boutron-Ruault MC, Clavel-Chapelon F. Estrogen-progestagen menopausal hormone therapy and breast cancer: does delay from menopause onset to treatment initiation influence risks? J Clin Oncol. 2009; 27:5138–43. https://doi.org/10.1200/jco.2008.21.6432.

18. Fournier A, Berrino F, Riboli E, Avenel V, Clavel-Chapelon F. Breast cancer risk in relation to different types of hormone replacement therapy in the E3N-EPIC cohort. International Journal of Cancer. 2005; 114:448–54.

19. Ritte R, Lukanova A, Berrino F, Dossus L, Tjonneland A, Olsen A, Overvad TF, Overvad K, Clavel-Chapelon F, Fournier A, Fagherazzi G, Rohrmann S, Teucher B, et al. Adiposity, hormone replacement therapy use and breast cancer risk by age and hormone receptor status: a large prospective cohort study. Breast Cancer Res. 2012; 14:R76. https://doi.org/10.1186/bcr3186.

20. Reeves GK, Beral V, Green J, Gathani T, Bull D, Million Women Study C. Hormonal therapy for menopause and breast-cancer risk by histological type: a cohort study and meta-analysis. Lancet Oncology. 2006; 7:910–8. https://doi.org/10.1016/s1470-2045(06)70911-1.

21. Beral V. Breast cancer and hormone-replacement therapy in the Million Women Study. Lancet. 2003; 362:419–27.

22. Saxena T, Lee E, Henderson KD, Clarke CA, West D, Marshall SF, Deapen D, Bernstein L, Ursin G. Menopausal hormone therapy and subsequent risk of specific invasive breast cancer subtypes in the California Teachers Study. Cancer Epidemiology Biomarkers and Prevention. 2010; 19:2366–78.

23. Kotsopoulos J, Chen WY, Gates MA, Tworoger SS, Hankinson SE, Rosner BA. Risk factors for ductal and lobular breast cancer: results from the nurses' health study. Breast Cancer Res. 2010; 12:R106. https://doi.org/10.1186/bcr2790.

24. Chen WY, Manson JE, Hankinson SE, Rosner B, Holmes MD, Willett WC, Colditz GA. Unopposed estrogen therapy and the risk of invasive breast cancer Nurses' Health Study. Archives of Internal Medicine. 2006; 166:1027–32.

25. Lyytinen H, Pukkala E, Ylikorkala O. Breast cancer risk in postmenopausal women using estradiol-progestogen therapy. Obstet Gynecol. 2009; 113:65–73. https://doi.org/10.1097/AOG.0b013e31818e8cd6.

26. Calle EE, Feigelson HS, Hildebrand JS, Teras LR, Thun MJ, Rodriguez C. Postmenopausal hormone use and breast cancer associations differ by hormone regimen and histologic subtype. Cancer. 2009; 115:936–45. https://doi.org/10.1002/cncr.24101.

27. Brinton LA, Richesson D, Leitzmann MF, Gierach GL, Schatzkin A, Mouw T, Hollenbeck AR, Lacey JV, Jr. Menopausal hormone therapy and breast cancer risk in the NIH-AARP Diet and Health Study Cohort. Cancer Epidemiol Biomarkers Prev. 2008; 17:3150–60. https://doi.org/10.1158/1055-9965.epi-08-0435.

28. Espié M, Daures JP, Chevallier T, Mares P, Micheletti MC, De Reilhac P. Breast cancer incidence and hormone replacement therapy: Results from the MISSION study, prospective phase. Gynecological Endocrinology. 2007; 23:391–7.

29. 2006 R, 2006 R, Rosenberg 2006Rosenberg L, Palmer JR, Wise LA, Adams-Campbell LL. A prospective

study of female hormone use and breast cancer among black women. Arch Intern Med. 2006; 166:760–5. https://doi.org/10.1001/archinte.166.7.760.

30. Tjonneland A, Christensen J, Thomsen BL, Olsen A, Overvad K, Ewertz M, Mellemkjaer L. Hormone replacement therapy in relation to breast carcinoma incidence rate ratios: a prospective Danish cohort study. Cancer. 2004; 100:2328–37. https://doi.org/10.1002/cncr.20250.
31. Stahlberg C, Pedersen AT, Lynge E, Andersen ZJ, Keiding N, Hundrup YA, Obel EB, Ottesen B. Increased risk of breast cancer following different regimens of hormone replacement therapy frequently used in Europe. International Journal of Cancer. 2004; 109:721–7.
32. Feigelson HS, Jonas CR, Teras LR, Thun MJ, Calle EE. Weight Gain, Body Mass Index, Hormone Replacement Therapy, and Postmenopausal Breast Cancer in a Large Prospective Study. Cancer Epidemiology Biomarkers and Prevention. 2004; 13:220–4.
33. Bakken K, Alsaker E, Eggen AE, Lund E. Hormone replacement therapy and incidence of hormone-dependent cancers in the Norwegian women and cancer study. International Journal of Cancer. 2004; 112:130–4.
34. Olsson HL, Ingvar C, Bladstrom A. Hormone replacement therapy containing progestins and given continuously increases breast carcinoma risk in Sweden. Cancer. 2003; 97:1387–92. https://doi.org/10.1002/cncr.11205.
35. Jernstrom H, Bendahl PO, Lidfeldt J, Nerbrand C, Agardh CD, Samsioe G. A prospective study of different types of hormone replacement therapy use and the risk of subsequent breast cancer: the women's health in the Lund area (WHILA) study (Sweden). Cancer Causes Control. 2003; 14:673–80.
36. de Lignieres B, de Vathaire F, Fournier S, Urbinelli R, Allaert F, Le MG, Kuttenn F. Combined hormone replacement therapy and risk of breast cancer in a French cohort study of 3175 women. Climacteric. 2002; 5:332–40.
37. Chen CL, Weiss NS, Newcomb P, Barlow W, White E. Hormone replacement therapy in relation to breast cancer. Journal of the American Medical Association. 2002; 287:734–41.
38. Manjer J, Malina J, Berglund G, Bondeson L, Garne JP, Janzon L. Increased incidence of small and well-differentiated breast tumours in post-menopausal women following hormone-replacement therapy. International Journal of Cancer. 2001; 92:919–22.
39. Schairer C, Lubin J, Troisi R, Sturgeon S, Brinton L, Hoover R. Menopausal estrogen and estrogen-progestin replacement therapy and breast cancer risk. Journal of the American Medical Association. 2000; 283:485–91.
40. Persson I, Weiderpass E, Bergkvist L, Bergstrom R, Schairer C. Risks of breast and endometrial cancer after estrogen and estrogen-progestin replacement. Cancer Causes Control. 1999; 10:253–60.
41. Lando JF, Heck KE, Brett KM. Hormone replacement therapy and breast cancer risk in a nationally representative cohort. American Journal of Preventive Medicine. 1999; 17:176–80.
42. Gapstur SM, Morrow M, Sellers TA. Hormone replacement therapy and risk of breast cancer with a favorable histology: Results of the Iowa Women's Health Study. Journal of the American Medical Association. 1999; 281:2091–7+141.
43. Sourander L, Rajala T, Raiha I, Makinen J, Erkkola R, Helenius H. Cardiovascular and cancer morbidity and mortality and sudden cardiac death in postmenopausal women on oestrogen replacement therapy (ERT). Lancet. 1998; 352:1965–9. https://doi.org/10.1016/s0140-6736(98)05066-1.
44. Schuurman AG, Van den Brandt PA, Goldbohm RA. Exogenous hormone use and the risk of postmenopausal breast cancer: Results from the Netherlands Cohort Study. Cancer Causes and Control. 1995; 6:416–24.
45. Risch HA, Howe GR. Menopausal hormone usage and breast cancer in Saskatchewan: a record-linkage cohort study. Am J Epidemiol. 1994; 139:670–83.
46. Mills PK, Beeson WL, Phillips RL, Fraser GE. Prospective study of exogenous hormone use and breast cancer in Seventh-day Adventists. Cancer. 1989; 64:591–7.
47. Adami HO, Persson I, Hoover R, Schairer C, Bergkvist L. Risk of cancer in women receiving hormone replacement therapy. International Journal of Cancer. 1989; 44:833–9.
48. Chlebowski RT, Anderson GL, Aragaki AK, Prentice R. Breast Cancer and Menopausal Hormone Therapy by Race/Ethnicity and Body Mass Index. Journal of the National Cancer Institute. 2016; 108.
49. Prentice RL, Chlebowski RT, Stefanick ML, Manson JE, Pettinger M, Hendrix SL, Hubbell FA, Kooperberg C, Kuller LH, Lane DS, McTiernan A, Jo O'Sullivan M, Rossouw JE, et al. Estrogen plus progestin therapy and breast cancer in recently postmenopausal women. American Journal of Epidemiology. 2008; 167:1207–16.
50. Luo J, Cochrane BB, Wactawski-Wende J, Hunt JR, Ockene JK, Margolis KL. Effects of menopausal hormone therapy on ductal carcinoma in situ of the breast. Breast Cancer Research and Treatment. 2013; 137:915–25.
51. Fournier A, Fabre A, Mesrine S, Boutron-Ruault MC, Berrino F, Clavel-Chapelon F. Use of different postmenopausal hormone therapies and risk of histology- and hormone receptor-defined invasive breast cancer. Journal Of Clinical Oncology. 2008; 26:1260–8. https://doi.org/10.1200/jco.2007.13.4338.
