# Supplementary material for: Change in risk of breast cancer after receiving hormone replacement therapy by considering effect-modifiers: a systematic review and dose-response meta-analysis of prospective studies
Source: Oncotarget. 2017 Aug 11;8(46):81109–24. doi: 10.18632/oncotarget.20154 (PMC5655266; doi:10.18632/oncotarget.20154)
Supplement: Supplementary file 2 [file oncotarget-08-81109-s002.docx]

**Supplementary TableS2. Reasons for exclusion of full manuscripts screened and not included in meta-analysis**

| No. | Title | Year | Reason* |
| --- | --- | --- | --- |
| 1 | Menopausal hormone therapy use and breast cancer risk in Australia: Findings from the New South Wales Cancer, Lifestyle and Evaluation of Risk study | 2016 | 2 |
| 2 | Interactions between breast cancer susceptibility loci and menopausal hormone therapy in relationship to breast cancer in the Breast and Prostate Cancer Cohort Consortium | 2016 | 14 |
| 3 | Time trends in breast cancer and menopause hormone therapy use in New Zealand | 2016 | 1 |
| 4 | Menopausal hormone therapy use in relation to breast cancer incidence in 11 European countries | 2016 | 1 |
| 5 | Hormonal contraceptives and hormone replacement therapy as a possible factor of breast cancer | 2015 | 9 |
| 6 | Hormone therapy and young-onset breast cancer | 2015 | 2 |
| 7 | Prediagnostic Sex Steroid Hormones in Relation to Male Breast Cancer Risk | 2015 | 6 |
| 8 | Sex hormone associations with breast cancer risk and the mediation of randomized trial postmenopausal hormone therapy effects | 2014 | 6 |
| 9 | Is breast cancer risk the same for all progestogens? | 2014 | 2 |
| 10 | Menopausal hormone therapy and breast cancer | 2014 | 3 |
| 11 | Menopausal hormone therapy (MHT) use and breast cancer risk in Australia: Initial findings from the NSW clear study | 2014 | 2 |
| 12 | Hormone therapy and risk of breast cancer | 2014 | 4 |
| 13 | Fatal breast cancer risk in relation to use of unopposed estrogen and combined hormone therapy | 2014 | 7 |
| 14 | Risk of breast cancer with hormone replacement therapy | 2014 | 3 |
| 15 | Use of menopausal hormone therapy and risk of ductal and lobular breast cancer among women 55-74 years of age | 2014 | 2 |
| 16 | Timing of oral contraceptive use and the risk of breast cancer in BRCA1 mutation carriers | 2014 | 5 |
| 17 | Interactions of hormone replacement therapy, body weight, and bilateral oophorectomy in breast cancer risk | 2014 | 2 |
| 18 | Breast cancer risk with hormone replacement after risk-reducing salpingo-oophorectomy in BRCA mutation carriers: Does it abrogate the benefit? | 2014 | 14 |
| 19 | Hormone Replacement Therapy and Breast Cancer: Heterogeneous Risks by Race, Weight, and Breast Density | 2013 | 1 |
| 20 | Sex hormone levels and risk of breast cancer with estrogen plus progestin | 2013 | 6 |
| 21 | Risk of breast cancer by type of menopausal hormone therapy: a case-control study among post-menopausal women in France | 2013 | 2 |
| 22 | Estrogen Plus Progestin and Breast Cancer Incidence and Mortality in the Women’s Health Initiative Observational Study | 2013 | 4 |
| 23 | Hormonal therapy and risk of breast cancer in Mexican women | 2013 | 2 |
| 24 | Hormone therapy use and breast cancer incidence by histological subtypes in Sweden and Norway | 2012 | 1 |
| 25 | Population-based hormone receptor-specific incidence trends of breast cancer in Germany | 2012 | 1 |
| 26 | Breast cancer risk during hormone therapy: experimental versus clinical data | 2012 | 1 |
| 27 | Postmenopausal hormone therapy is associated with in situ breast cancer risk | 2012 | 3 |
| 28 | Menopause-associated symptoms, HRT use and risk of young-onset breast cancer: Results from the Two Sister Study | 2012 | 2 |
| 29 | Postmenopausal hormone therapy and ductal carcinoma in situ: a population-based case-control study | 2012 | 2 |
| 30 | Hormone replacement therapy and the risk of breast cancer | 2011 | 3 |
| 31 | Effect of menopausal hormone therapy on risk of ductal or lobular breast cancer and tumor characteristics | 2011 | 2 |
| 32 | Hormone replacement therapy and breast cancer | 2011 | 3 |
| 33 | Breast cancer risk and hysterectomy status: The Multiethnic Cohort study | 2009 | 1 |
| 34 | Breast cancer risk factors in Turkish women--a University Hospital based nested case control study | 2009 | 5 |
| 35 | Influence of estrogen plus testosterone supplementation on breast cancer | 2009 | 4 |
| 36 | Hormone replacement therapy use and variations in the risk of breast cancer | 2008 | 2 |
| 37 | Interactions between intakes of alcohol and postmenopausal hormones on risk of breast cancer | 2008 | 11 |
| 38 | Health risks and benefits 3 years after stopping randomized treatment with estrogen and progestin | 2008 | 1 |
| 39 | Unequal risks for breast cancer associated with different hormone replacement therapies: results from the E3N cohort study | 2008 | 4 |
| 40 | Use of oral conjugated estrogen alone and risk of breast cancer | 2007 | 1 |
| 41 | The effect of mammographic screening and hormone replacement therapy use on breast cancer incidence in England and Wales | 2007 | 1 |
| 42 | Estrogen affects post-menopausal women differently than estrogen plus progestin replacement therapy | 2007 | 1 |
| 43 | Hormone replacement therapy and breast cancer in former users of oral contraceptives--The Norwegian Women and Cancer study | 2007 | 12 |
| 44 | Breast tumours following combined hormone replacement therapy express favourable prognostic factors | 2007 | 1 |
| 45 | Effects of Conjugated Equine Estrogens on Breast Cancer and Mammography Screening in Postmenopausal Women with Hysterectomy | 2006 | 15 |
| 46 | Breast cancer in postmenopausal women with and without hormone replacement therapy: Preliminary results of the MISSION study | 2006 | 4 |
| 47 | Prior hormone therapy and breast cancer risk in the Women's Health Initiative randomized trial of estrogen plus progestin | 2006 | 4 |
| 48 | Breast cancer incidence, case-fatality and breast cancer mortality in Danish women using hormone replacement therapy - A prospective observational study | 2005 | 4 |
| 49 | Breast cancer with different prognostic characteristics developing in Danish women using hormone replacement therapy | 2004 | 7 |
| 50 | Impact of hormone replacement therapy (HRT) on incidence of breast cancer in Norway - The NOWAC study | 2004 | 4 |
| 51 | Prognostic characteristics of breast cancer among postmenopausal hormone users in a screened population | 2003 | 1 |
| 52 | Breast cancer in a multiethnic cohort in Hawaii and Los Angeles: risk factor-adjusted incidence in Japanese equals and in Hawaiians exceeds that in whites | 2002 | 5 |
| 53 | Endogenous sex hormones and breast cancer in postmenopausal women: reanalysis of nine prospective studies | 2002 | 6 |
| 54 | Incidence of cardiovascular disease, cancer and death in postmenopausal women affirming use of hormone replacement therapy | 2002 | 1 |
| 55 | Use of postmenopausal hormones, alcohol, and risk for invasive breast cancer | 2002 | 11 |
| 56 | Cumulative risk of breast cancer to age 70 years according to risk factor status: data from the Nurses' Health Study | 2000 | 5 |
| 57 | The role of hormone replacement therapy in the risk for breast cancer and total mortality in women with a family history of breast cancer | 1997 | 13 |
| 58 | Estrogen replacement therapy and risk of fatal breast cancer in a prospective cohort of postmenopausal women in the United States | 1997 | 7 |
| 59 | Post-menopausal hormone therapy and mortality | 1996 | 7 |
| 60 | Incidence of benign and malignant breast disorders in women taking hormones (contraceptive pill or hormonal replacement therapy) | 1996 | 2 |
| 61 | Long-term hormone replacement therapy and risk of breast cancer in postmenopausal women | 1995 | 2 |
| 62 | Oestrogens and progestins and breast cancer risk in post-menopausal women | 1995 | 3 |
| 63 | The use of oestrogens and progestin and the risk of breast cancer in post-menopausal women. | 1995 | 8 |
| 64 | Hormonal replacement therapy and morbidity and mortality in a prospective study of postmenopausal women | 1995 | 4 |
| 65 | Menopausal estrogen and estrogen-progestin replacement therapy and risk of breast cancer (United States) | 1994 | 4 |
| 66 | Hormone replacement therapy and breast cancer mortality in Swedish women: Results after adjustment for 'healthy drug-user' effect | 1993 | 7 |
| 67 | Swedish studies link hormone use to higher breast cancer risk | 1989 | 8 |
| 68 | Influence of exogenous estrogens, proliferative breast disease, and other variables on breast cancer risk | 1989 | 9 |
| 69 | Long-term surveillance of mortality and cancer incidence in women receiving hormone replacement therapy | 1987 | 2 |
| 70 | Use of progestogen therapy | 1987 | 3 |
| 71 | Hormones in the etiology and prevention of breast and endometrial cancer | 1984 | 1 |
| 72 | Decreased incidence of breast cancer in postmenopausal estrogen-progestogen users | 1983 | 1 |
| 73 | Minimal dose combined hormonal replacement therapy for menopausal women | 1982 | 10 |
| 74 | Estrogen therapy and breast cancer in postmenopausal women | 1981 | 1 |
| 75 | Menopausal estrogens and breast cancer | 1976 | 1 |
| 76 | The effects of long-term estrogen on hysterectomized women | 1974 | 7 |
| 77 | Surgically confirmed gallbladder disease, venous thromboembolism, and breast tumors in relation to postmenopausal estrogen therapy. A report from the Boston Collaborative Drug Surveillance Program, Boston University Medical Center | 1974 | 1 |
| Abbreviations: OR, odds ratio; RR, relative risk; HR, hazard ratio.  * Reasons for exclusion classified as: 1-Not reporting adjusted OR/RR/HR(n=19); 2- Case-control studies (n=15); 3-Reviews(n=7); 4-Duplicate studies on the same study population without valid data on their contribution(n=7); 5-Irrelevant to HRT(n=4); 6-Exposure for serum Sex hormones(n=4); 7-Reporting the association of HRT use and BC death risk(n=5); 8-Editorials(n=2); 9- Retrospective cohort studies(n=2); 10- Irrelevant to breast cancer(n=2); 11-Outcomes interacted with alcohol(n=2); 12-Interacted with oral contraceptives consumption(n=1); 13-Subjects with a family history of breast cancer(n=1); 14-Outcomes interacted with breast cancer susceptibility loci(n=1); 15- Reporting the association of mammography screening and BC risk after HRT use(n=1). | | | |
