# Supplementary material for: Change in risk of breast cancer after receiving hormone replacement therapy by considering effect-modifiers: a systematic review and dose-response meta-analysis of prospective studies
Source: Oncotarget. 2017 Aug 11;8(46):81109–24. doi: 10.18632/oncotarget.20154 (PMC5655266; doi:10.18632/oncotarget.20154)
Supplement: Supplementary file 3 [file oncotarget-08-81109-s003.docx]

**Supplementary TableS3 Characteristics of included studies.**

| **Study(Year); Location** | **Study design** | **Cohort/Data source** | **Mean follow-up (years)** | **Mean**  **Age (years)** | **Exposure (interventional) variables** | **Cases/Sample size** | **Ascertainment of cases** | **Adjustments factors** |
| --- | --- | --- | --- | --- | --- | --- | --- | --- |
| Roman et al (2016);  Norway[^5^](#_ENREF_5) | PCS | Norwegian prescription database | 4.8 | 59.4 | ET; EPT; tibolone | 7,910/686,614 | Pathological proving | Age, age at FB, NOB. |
| Jones et al (2016);  UK[^6^](#_ENREF_6) | PCS | Breakthrough Generations Study (GBS) | 6 | 57 | ET; EPT | 775/39,183 | Cancer registries | Attained age and AAM. |
| Suhrke et al  (2015); Norway[^7^](#_ENREF_7) | PCS | Norwegian breast cancer screening program(NBCSP) | 5 | NR | ET; EPT; tibolone | 5,278/449,717 | Cancer registries | Age, NOB, whether or not the women attended the screening program in 2004–2009, whether or not a nonuser in 2004–2005 started with HT use in 2006–2009. |
| Chlebowski et al  (2015);  USA[^2^](#_ENREF_2) | RCT | Women’s Health Initiative (WHI) | 13 | 63.4 | ET; EPT | NR/27,347 | Medical record | NA. |
| Chlebowski et al  (2015); USA[^8^](#_ENREF_8) | RCT | Women’s Health Initiative  (WHI) | 6.2 | NR | ET; EPT | 1,141/27,347 | Medical record | NA. |
| Luo et al (2013); USA[^9^](#_ENREF_9) | RCT and PCS | Women’s Health Initiative (WHI) | 5.6/7.1^a^ | NR | ET; EPT | 265/26,463 | Medical record | Age, race, education, FH, Gail 5-year risk, AAM, parity/age at FB, OC use，BMI, smoking status, alcohol intake, physical activity, pregnancy. |
| Anderson et al (2012); USA[^10^](#_ENREF_10) | RCT and PCS | Women’s Health Initiative (WHI) | 11.8 | 63.4 | ET | 350/10,739 | Medical and  pathological record | Age, race, education, FH, marital status, BMI, smoking status, AAM, number of term pregnancies, age at FB, number of months breastfed, benign breast disease, Gail model 5-year risk score, bilateral oophorectomy, age at hysterectomy. |
| LaCroix et al (2011); USA[^11^](#_ENREF_11) | RCT and PCS | Women’s Health Initiative  (WHI) | 10.7 | NR | ET | 350/10,739 | Medical record | Age, race, HRT use status, BMI, smoking status, parity/age at FB, hysterectomy age group, bilateral oophorectomy, history of CABG/PTCA, treated diabetes, hypertensive, history of high cholesterol requiring pills, statin group, history of angina, stroke ever. |
| Prentice et al (2008); USA[^12^](#_ENREF_12) | RCT and PCS | Women’s Health Initiative  (WHI) | 5.6 | 63.2 | EPT | 172/16,608 | Medical record，pathological reports | Age, Gail 5-year risk, BMI, FH of BC, and prior diagnosis of BBD. |
| Anderson et al (2004); USA[^13^](#_ENREF_13) | RCT | Women’s Health Initiative  (WHI) | 6.8 | NR | ET | 218/10,739 | Medical record | Age at screening, race, duration of prior hormone use, BP, age at FB, smoking status, parity/age at hysterectomy, medical treatment, bilateral oophorectomy, medical history, fracture at age. |
| Rossouw et al (2002); USA[^14^](#_ENREF_14) | RCT | Women’s Health Initiative  (WHI) | 5.2 | 63.2 | EPT | 166/16,608 | Pathological reports | Age, race, duration of prior hormone use, BMI, BP, smoking status, parity/age at FB, treated for diabetes or hypertension or elevated cholesterol levels, statin use at baseline, aspirin use at baseline, history of myocardial infarction or angina or stroke, history of CABG/PTCA, history of DVT or PE, FH of BC, fracture at age, Gail 5-year risk. |
| Thorbjarnard-ottir et al  (2014);  Iceland[^15^](#_ENREF_15) | PCS | Icelandic Cancer Detection Clinic (CDC) | 7.6 | 59.2 | ET; EPT | 315/16,928 | NR | Birth year, age when giving information, AAM, NOB, AAFB. |
| Fournier et al  (2014);  French[^16^](#_ENREF_16) | PCS | French E3N Cohort | 11.2 | 66 | ET; EPT; tibolone | 3,678/78,353 | Pathology reports | Birth year, parity/age at FB, BMI, menopausal type, menarche year, BC history in relatives, MHT exposure. |
| Fournier et al (2009); French[^17^](#_ENREF_17) | PCS | French E3N Cohort | 8.1 | 54.6 | EPT | 1,726/53,310 | Pathological reports | Age (time scale) and AAM (continuous). |
| Fournier et al (2005); French[^18^](#_ENREF_18) | PCS | French E3N cohort study | 5.8 | 52.8 | ET; EPT | 948/54,548 | Pathology reports | Time since menopause, BMI, pregnancy at age, FH of BC, personal history of BBD, use of oral progestogens before menopause, ever use of OC and previous mammography. |
| Ritte et al (2012); European  countries[^19^](#_ENREF_19) | PCS | European Prospective Investigation into  Cancer and Nutrition (EPIC) | 10.8 | 50.9 | ET; EPT | 9,530/314,676 | Pathology registries | Study center, age, height, educational attainment, smoking status, alcohol consumption, parity/age at FB, AM, menopausal status, AAM, ever OC use and current HRT use. |
| Lai et al (2011);  Taiwan[^3^](#_ENREF_3) | PCS | National Health Insurance (NHI) of Taiwan | 9 | 40.6 | ET; EPT | 2,461/65,723 | National Health Insurance (NHI) catastrophic illnesses registry | NR |
| Beral et al (2010);  UK[^1^](#_ENREF_1) | PCS | Million Women Study | 4 | 56.6 | ET; EPT; tibolone | 15,759/  1,129, 025 | Cancer registry | Region of residence, socioeconomic  status, AAM, BMI, parity/age at FB, alcohol consumption. |
| Reeves et al (2006); UK[^20^](#_ENREF_20) | PCS | Million Women Study | 3.5 | NR | ET; EPT | 14,102/  1,031,124 | Cancer registry | Region, parity/age at FB, time since menopause, deprivation index, BMI, and FH of BC. |
| Beral et al (2003);  UK[^21^](#_ENREF_21) | PCS | Million Women Study | 2.6 | NR | ET; EPT | 9,364/  1,084,110 | Cancer registry | Age, time since menopause, parity/age at FB, FH of BC, BMI, region, and deprivation index. |
| Saxena et al  (2010);  USA[^22^](#_ENREF_22) | PCS | California Teachers Study (CTS) | 9.8 | 60.9 | ET; EPT | 2,857/56,867 | California Cancer Registry (CCR) | Race, FH of BC, BMI, smoking, alcohol consumption, mammographic screening, parity and age at full-term pregnancy, AAM, AM, and history of breast biopsy. |
| Kotsopoulos et al (2010);  USA[^23^](#_ENREF_23) | PCS | Nurses’ Health Study | 16.5 | 54.5 | ET; EPT | 62,26/107,759 | Medical records | Age, AM, parous  nulliparous, parity/age at FB, postmenopausal hormone use, AAM,  Menopausal. |
| Chen et al (2006); USA[^24^](#_ENREF_24) | PCS | Nurses’ Health Study | 11.6 | 59.3 | ET | 934/1,6041 | Pathology reports or cancer  registry. | Age, AAM, AM, BMI, history of BBD, FH of breast cancer in first-degree relative, average daily alcohol consumption, parity/age at FB. |
| Lyytinen et al  (2009); Finland[^25^](#_ENREF_25) | PCS | National Medical Reimbursement Register | 6.88 | 55.6 | EPT | 6,211/221,551 | Finnish Cancer Registry | NR. |
| Calle et al (2009);  USA[^26^](#_ENREF_26) | PCS | Cancer Prevention Study II (CPS-II) Nutrition Cohort | 13 | 62.4 | ET; EPT | 1,821/67,754 | Medical records or state cancer registry | Age at 1992 survey, hysterectomy and oophorectomy, time-dependent mammography, education, BMI, type of menopause, and AAM. |
| Brinton et al (2008);  USA[^27^](#_ENREF_27) | PCS | NIH-AARP Diet and Health Study Cohort | 5.3 | 62.6 | ET; EPT | 3,657/126,638 | Cancer registry | Age at study entry, parity, race, AAFB, menopausal status, number of breast biopsies, FH of BC, and number of mammograms. |
| Espie et al (2007);  French[^28^](#_ENREF_28) | PCS | MISSION Study (Menopause: Risk of Breast Cancer, Morbidity and Prevalence) | 8.3 | 62.2 | ET; EPT | 31/4,949 | NR | Age. |
| Rosenberg et al  (2006);  USA[^29^](#_ENREF_29) | PCS | Black Women’s Health Study (BWHS). | 7.84 | 49.8 | ET; EPT | 615/23,191 | Medical records | Age at baseline in 1-year intervals, menopausal status including premenopausal, naturally menopausal, bilateral oophorectomy, and hysterectomy, and age at menopause or hysterectomy |
| Ewertz et al (2005); Denmark[^4^](#_ENREF_4) | PCS | County of North Jutland, Denmark, and the Danish Cancer Registry | 10 | NR | ET; EPT | 1,462/78,380 | Cancer registry | Calendar period and number of children and age at FB. |
| Tjonneland et al (2004);  Denmark[^30^](#_ENREF_30) | PCS | Diet, Cancer and Health Study | 4.8 | 57 | EPT | 423/23,618 | Cancer Registry | Duration of therapy and time since last use, the presented rate ratios compare current users registered at baseline with participants who never received HRT. |
| Stahlberg et al (2004);  Denmark[^31^](#_ENREF_31) | PCS | Danish Nurse Cohort | 6.34 | 59.7 | ET; EPT;  tibolone | 244/10,874 | Cancer Registry | Age, benign breast disease and AAM. |
| Feigelson et al (2004); USA[^32^](#_ENREF_32) | PCS | Cancer Prevention Study (CPS)-II Nutrition Cohort | 7 | 62.7 | Mixed HRT | 1,934/62,756 | Medical records | Age at interview, AM, AAM, number of live births, AAFB, OC use, FH of BC, history of mammography, height, education, physical activity, alcohol use, and race, total adult weight gain. |
| Bakken et al  (2004);  Norway[^33^](#_ENREF_33) | PCS | Norwegian Woman and Cancer Study (NOWAC) | 8 | 53 | EPT | 624/31,451 | NR | Age, BMI, AM, ever use of OCs, time since menopause, FH of BC, mammography,  parity/age at FB. |
| Olsson et al  (2003);  Sweden[^34^](#_ENREF_34) | PCS | South Swedish Regional and National Swedish Tumour Registry | 10.12 | NR | ET; PT; EPT | 556/28,378 | The population-based census  registry, cause of death registry, and the swedish  cancer registry | Each factor simultaneously, year of interview menopause age. |
| Jernstrom et al (2003);  Sweden[^35^](#_ENREF_35) | PCS | Women's Health in the Lund Area (WHILA) | 4.1 | 56.4 | EPT | 101/6,586 | The South Swedish tumor registry | Age at study entry. |
| Lignieres et al (2002);  France[^36^](#_ENREF_36) | PCS | French ERN Cohort | 8.9 | 50 | ET; EPT | 105/3,175 | Local cancer registries | Period of treatment, AAFB and AAM. |
| Chen et al (2002);  USA[^37^](#_ENREF_37) | NCC-S | Group Health Cooperative of Puget Sound (GHC) | 5 | NR | ET; EPT | 692/1,334 | Pathological proving | Age at reference, year of diagnosis, and number of mammograms before diagnosis. |
| Manjer et al  (2001);  Sweden[^38^](#_ENREF_38) | PCS | Sweden Cohort | 9.8 | 54.1 | Mixed HRT | 141/5,865 | The swedish cancer  registry and local clinical registries | Age at baseline, height, BMI, AM, nulliparity, education and smoking habits. |
| Schairer et al  (2000);  USA[^39^](#_ENREF_39) | PCS | Breast Cancer Detection Demonstration Project (BCDDP) | 10.2 | 58 | ET; PT; EPT | 2,082/46,355 | Self-reports or reports of breast cancer on death certificates | Age, follow-up time, age at first full-term pregnancy, BMI, education, AAM. |
| Persson et al  (1999);  Sweden[^40^](#_ENREF_40) | PCS | National Swedish Cancer Registry | 5.7 | 65 | ET; EPT | 198/10,472 | The national swedish cancer  registry | Age, follow-up time, age at first full-term pregnancy, BMI, education, AAM. |
| Lando et al  (1999);  USA[^41^](#_ENREF_41) | PCS | The First National Health and Nutrition Examination Survey (NHANES I) | 12.7 | 55.5 | Mixed HRT | 3,564/5,761 | NR | Age, race, education, BMI, AAFB AAM, type  of menopause, and FH of BC. |
| Gapstur et al  (1999);  USA[^42^](#_ENREF_42) | PCS | Iowa Women’s Health Study | 11 | NR | Mixed HRT | 1,164/37,105 | Pathological proving | Age, BMI, BMI at age 18 years, waist-to-hip ratio, AM, AAM, AAFB, parity, FH of BC in a first-degree relative, type  of menopause, and alcohol intake. |
| Sourander et al (1998); Finland[^43^](#_ENREF_43) | PCS | Mammography Screening for breast cancer | 6.7 | 60 | ET | 5,572/7,944 | Pathological proving | NR. |
| Schuurman et al (1995);  Netherlands[^44^](#_ENREF_44) | PCS | Netherlands Cohort Study (NLCS) | 3.3 | NR | Mixed HRT | 471/62,573 | Pathological proving | Age, benign breast disease, mother with BC, sister(s) with BC, parity/age at FB, AM, AAM, induced menopause, education, current cigarette smoking, BMI, alcohol use, energy consumption, and use of OCs. |
| Risch et al (1994); Canada[^45^](#_ENREF_45) | PCS | Saskatchewan Health Prescription-Drug-Plan Database | 11 | NR | ET; EPT | 742/32,790 | NR | NR. |
| Mills et al (1989);  USA[^46^](#_ENREF_46) | PCS | Seventh-day Adventist Cohort | 6 | 55.4 | Mixed HRT | 153/2,0341 | Pathological proving | NR. |
| Adami et al (1989);  Sweden[^47^](#_ENREF_47) | PCS | Uppsala health care region | 6.7 | 54.5 | ET | 300/23,244 | The national cancer registry | NR. |

**Note:** Items with gray context are overlapping publications with detailed information, not for main analyses (i.e. categorical analyses, dose-response meta-analyses), were included and exploited for subgroup analyses**.** Seven eligible publications[^2^](#_ENREF_2)^,^ [^11^](#_ENREF_11)^,^ [^13^](#_ENREF_13)^,^ [^14^](#_ENREF_14)^,^ [^48-50^](#_ENREF_48) reported results from WHI RCT and its subsequent observational study. Four eligible publications[^16-18^](#_ENREF_16)^,^ [^51^](#_ENREF_51) reported results from E3N cohort, and three [^1^](#_ENREF_1)^,^ [^20^](#_ENREF_20)^,^ [^21^](#_ENREF_21)reported on Million Women Study. Nurses’ Health Study cohort was effectively reported by two publications[^23^](#_ENREF_23)^,^ [^24^](#_ENREF_24).

**^a^ The average follow-up for the WHI CEE+MPA and WHI CEE-alone trial was 5.6 and 7.1 years, respectively**

**Abbreviations: PCS, prospective cohort study;** **ET, estrogen-only therapy; PT, progestin-only therapy; EPT, estrogen-progestin therapy; CEE, conjugated equine estrogens; MPA, medroxyprogesterone acetate; WHI, Women Health Initial study; NCCS, nested case-control study; FB, first birth;** **NOB, number of child births;** **AAM, age at menopause; AM, age at menarche;** **RCT: randomized controlled trail; NA, not associate; NR, not report; HRT, hormone replacement therapy; BMI, body mass index; FH, family history; OC, oral contraceptive; BP, blood Pressure; DVT, deep vein thrombosis; PE, pulmonary embolism; CABG/PTCA, coronary artery bypass grafting/percutaneous transluminal coronary angioplasty; BC, breast cancer; BBD, benign breast disease.**
